# Effectiveness of Acupuncture for Anxiety Among Patients With Parkinson Disease: A Randomized Clinical Trial

**DOI:** 10.1001/jamanetworkopen.2022.32133

**Published:** 2022-09-21

**Authors:** Jing-qi Fan, Wei-jing Lu, Wei-qiang Tan, Xin Liu, Yu-ting Wang, Nan-bu Wang, Li-xing Zhuang

**Affiliations:** 1 Guangzhou University of Chinese Medicine, Guangzhou 510006, Guangdong, China; 2 The First Affiliated Hospital of Guangzhou University of Chinese Medicine, Guangzhou 510000, Guangdong, China

## Abstract

**Importance:**

One of the ordinary manifestations of Parkinson disease (PD) is anxiety, which remains untreated. Anxiety is closely associated with the accelerated progression of PD. Efficacy of acupuncture for anxiety has been reported. However, to date, there are no data on acupuncture’s effectiveness on anxiety for patients with PD.

**Objective:**

To investigate the effect of acupuncture vs sham acupuncture for treating anxiety in patients with PD.

**Design, Setting, and Participants:**

This is randomized, double-blinded, clinical trial enrolled patients between June 20, 2021, and February 26, 2022. Final follow-up was April 15, 2022. Patients with Parkinson disease and anxiety were allocated randomly (1:1) to receive acupuncture or sham acupuncture for 8 weeks. Acupuncture operators, outcome measures evaluators, and statistical analysts were blinded to the grouping of patients. Patients were blinded to their own grouping during the study. This study took place in the Parkinson clinic of a hospital in China.

**Interventions:**

Real acupuncture or sham acupuncture for 8 weeks.

**Main Outcomes and Measures:**

Primary outcome was Hamilton Anxiety Scale (HAM-A) score. Secondary outcomes were scores on the Unified Parkinson Disease Rating Scale (UPDRS), 39-item Parkinson Disease Questionnaire (PDQ-39), and serum levels of the adrenocorticotropic hormone (ACTH) and cortisol (CORT).

**Results:**

Seventy eligible patients were enrolled, including 34 women (48.5%) and 36 men (51.4%). Sixty-four patients (91%) completed the intervention and the 8-week follow-up, including 30 women (46.9%) and 34 men (53.1%) with a mean (SD) age of 61.84 (8.47) years. At the end of treatment, the variation of HAM-A score was 0.22 (95% CI, –0.63 to 1.07; *P* = .62) between the real acupuncture and sham acupuncture groups. At the end of follow-up, the real acupuncture group had a significant 7.03-point greater (95% CI, 6.18 to 7.88; *P* < .001) reduction in HAM-A score compared with the sham acupuncture group. Four mild adverse reactions occurred during the study.

**Conclusions and Relevance:**

This study found acupuncture to be an effective treatment for anxiety in patients with PD. These findings suggest that acupuncture may enhance the wellbeing of patients who have Parkinson disease and anxiety.

**Trial Registration:**

Chinese Clinical Trial Registry: ChiCTR2100047253

## Key Points

**Question:**

What is the efficacy of acupuncture in treating patients who have Parkinson disease and anxiety?

**Findings:**

In this randomized clinical trial including 64 patients with Parkinson disease and anxiety who underwent 8 weeks of acupuncture with an 8-week follow-up, real acupuncture and sham acupuncture with clinical monitoring both significantly ameliorated anxiety at the end of treatment. However, real acupuncture significantly ameliorated anxiety at 2 months after treatment but sham acupuncture did not.

**Meaning:**

This study’s results suggest that acupuncture with clinical monitoring may alleviate anxiety of patients with Parkinson disease.

## Teaser

This randomized clinical trial investigates the effectiveness of acupuncture vs sham acupuncture for treating anxiety in patients with Parkinson disease.

## Introduction

Among the nonmotor symptoms of Parkinson disease (PD) is anxiety, which is generally untreated and usually evident in patients with PD. Anxiety in PD is identified by lack of concentration, continuous feeling of worry, muscle tension, and increased severity of tremors.^1^ Although often ignored, approximately 31% of patients with PD manifest symptoms of anxiety.^2^ Treatment, etiology, or symptoms of PD make anxiety a natural outcome (eFigure 1 in Supplement 2).^3^ Patients with PD and anxiety show greater disability and poorer wellbeing than these patients without anxiety.^4^ Moreover, disturbances in gait and freezing of gait have been reported to be associated with anxiety symptoms. Thus, anxiety should be regarded as a substantial symptom of PD associated with movement disorders.^5^ However, there are a limited number of methods specifically developed to deal with anxiety in PD.^6^ Medications such as dopamine agonists and antianxiety drugs may confer a mood benefit; however, their clinical effects appear to be small.^7,8^ Cognitive behavioral therapy (CBT) for anxiety in PD is promising.^9^ However, its high price leads to reduced compliance among patients. There is evidence that acupuncture is comparable to CBT.^10^ Considering shortcomings of existing therapies for anxiety in PD, the desire to explore effective alternative approaches with high feasibility and few adverse effects is growing in Western societies.^11^

Acupuncture has been recommended as a complementary and alternative therapy for neuropsychiatric symptoms of PD with level B evidence.^12,13,14^A systematic evaluation of 42 clinical studies showed that acupuncture with anti–Parkinson drug therapy has better effect on PD than anti–Parkinson drug therapy alone with fewer adverse effects.^12^ Acupuncture aimed at alleviating anxiety has clinical effects with good compliance.^15^ Nevertheless, we could not find data to confirm acupuncture’s utility for anxiety in PD. Thus, we conducted this double-blinded, clinical trial to investigate the effect of acupuncture for anxiety in PD. Our hypothesis is that acupuncture may prove beneficial in alleviating anxiety disturbances of patients with PD.

## Methods

### Study Design

This study was a randomized, double-blinded clinical trial. The participants were patients with PD and anxiety who were randomized 1:1 into an intervention and control group. Patients in both groups received clinical monitoring (CM). In the intervention group, patients received real acupuncture (RA), whereas patients in the control group received sham acupuncture (SA). The protocol was approved by the Ethics Committee of Guangzhou University of Traditional Chinese Medicine and monitored by an independent data and safety monitoring board (Supplement 1). Before initiation of this inquiry, all participants gave written informed consent, according to the guidelines of the Declaration of Helsinki. The medical ethics committees of all the included study centers provided their permission for the conduction of the current clinical trial. This study followed the Consolidated Standards of Reporting Trials (CONSORT) reporting guideline and the Standards for Reporting Interventions in Clinical Trials of Acupuncture (STRICTA) guideline for the designing and reporting of this trial. It was registered (ChiCTR2100047253) before inclusion of the first participant and its protocol has been publicly available.^3^

### Participants

Participants from the Parkinson clinic of the First Affiliated Hospital of Guangzhou University of Traditional Chinese Medicine were enrolled in the study after meeting the following criteria: (1) patients, diagnosed with idiopathic PD^16^; (2) PD diagnoses from 1 to 4 given according to Hoehn and Yahr scale; (3) patients’ anxiety assessed following the Hamilton Anxiety Scale (HAM-A) scores ranging from 14 to 29; (4) patients able to sign informed consent; (5) patients aged 35 to 80 years. The exclusion criteria were: (1) major cognitive impairment diagnosed following the Montreal Cognitive Assessment, score less than 23; (2) irresponsiveness to treatment with high doses of levodopa; (3) drug or alcohol misuse; (4) received acupuncture treatment within 30 days before treatment; (5) took antianxiety drugs within 30 days before treatment; (6) major neurologic, renal, cardiovascular, or hepatic deficiency; and (7) intolerance to acupuncture. Dropout criteria were as follows: (1) if participants’ anxiety symptoms worsened, it was recommended that the patient take antianxiety drugs after evaluation by a psychologist; (2) due to the aggravation of the patient’s condition during the treatment, it was recommended that the patient needed to change levodopa equivalent dose^17^ of anti–Parkinson drugs after the evaluation of neurological physicians.

### Randomization and Blinding

The participants were randomly allocated into the RA and SA groups in a ratio of 1:1. Randomization was created by a mathematician, who was not involved in the study, using SPSS Statistics version 26.0 (IBM).

Double-blinded acupuncture intervention using a special acupuncture tool was applied. In this study, some acupoints were penetrated at a 15° position. To date no sham needles are able to penetrate the skin at this angle. Consequently, we autonomously established a sham needle device, that could penetrate the acupoint from different angles on the basis of fixation. A patent has been obtained for this technology under the State Intellectual Property Office of China (authorization number: 202121352221.7). The sham acupuncture device is depicted in eFigure 3, eFigure 4, and eFigure 5 in Supplement 2. The masking effect of sham needle was shown in the eAppendix, eTable 4, and eTable 5 in Supplement 2. The tools used for patients in the 2 groups were identical in shape. In addition, duration of acupuncture and the acupoints used for the 2 groups were the same. Both groups of patients wore eye masks throughout the treatment to ensure the implementation of blinding. To achieve a double-blind outcome, after the operation assistants fixed the acupuncture auxiliary device on the acupoint skin; the acupuncture operators quickly tapped the top of the tube to make the needle go downward. Therefore, acupuncture operators were blinded to group and intervention by entering the needle through a tube. All patients’ status evaluations were done by a psychologist who was not aware of the study design and who did not know the patients’ classification.

### Intervention

All patients in the RA and SA groups received CM and maintained the original doses of their anti-Parkinson medications. If the medication needed to be changed, the observer recorded and calculated whether the equivalent dose of levodopa (eTable 5 in Supplement 1) changed as well.

### Clinical Monitoring

CM was performed with common leaflets depicting the patients’ ability to deal with nervousness. The questions were taken from web portals, maintained by psychiatric associations in France and the Netherlands.^9^

### Real and Sham Acupuncture

Participants in the RA and SA groups received 30-minute acupuncture once per day, 3 times per week for a period of 8 weeks, with fixed prescriptions according to the traditional Chinese medicine theory and information in previous articles on PD and anxiety.^15,18,19^ All participants received acupuncture at GV 24 (shen ting), GV 29 (yin tang), bilateral HT7 (shen men), bilateral SP 6 (san yin jiao), and Si Shen Zhen, which included 4 acupoints, including GV 21, GV 19, and 1.5 cun next to GV 20 bilaterally (eFigure 2 in Supplement 2). The names and locations of acupoints are labeled following the National Standard of the People’s Republic of China (GB/T 12346–2006), established in 2006.For RA, acupuncture was performed using one-use, sterile, stainless steel needles (Tianxie, Suzhou Medical Appliance Factory, Suzhou, China; 0.25 × 25 mm, 0.25 × 40 mm). Acupuncture operation process and acupoints was shown in eTable 3 in Supplement 2). For SA, acupuncture was performed using special disposable, sanitized, sham stainless steel needles. The participants in the SA group underwent a noninsertion procedure applied at the same acupoints and using the method as in the RA group. After needle insertion, the needle was twisted for 1 minute at a frequency of 180 to 200 rpm in both groups. After acupuncture was started, the needle was kept in place (in the skin) for 30 minutes in both groups.

### Assessments

Demographic and disease-related information of the participants were recorded at baseline. Since the patients were taking different types of anti-Parkinson drugs, the doses of the medications were converted to their levodopa equivalent daily doses.^17^ eTable 2 in Supplement 2 shows levodopa equivalent dose conversion. eTable 1 in Supplement 2 shows a full overview of the primary and secondary outcomes evaluated.

### Primary Outcome

The primary outcome was the Hamilton Anxiety Scale (HAM-A score), which was used for assessing the degree of anxiety. It consists of 14 symptomatic definition elements, with a total possible score of 56.

### Secondary Outcomes

The Unified Parkinson Disease Rating Scale (UPDRS) and the 39-item Parkinson Disease Questionnaire (PDQ-39) as well as the blood serum levels of ACTH (adrenocorticotropic hormone) and CORT (cortisol) were the secondary outcomes. Serum CORT and ACTH levels were evaluated by enzyme-linked immunosorbent assay (ELISA). The methods of serum preparation and ELISA operation are provided in Supplement 1.

### Assessment Time Points

HAM-A, UPDRS, and PDQ-39 scores were assessed at baseline, after treatment, and 8 weeks after treatment. Serum levels of ACTH and CORT were measured before and after treatment.

Due to symptom fluctuations and on-off phenomenon caused by medication, the results of scale evaluations may vary due to differences in the time medication was taken. Therefore, the scale evaluations and blood sampling were performed approximately 4 hours after medications were taken.

### Sample Size

The sample size was determined by the variation in the HAM-A score in pilot study. The mean (SD) HAM-A score of patients who received acupuncture combined with anti-Parkinson drugs was 15.3 (2.55), whereas that of patients who received the sham acupuncture with anti-Parkinson drugs was 13.2 (3.18). The power of statistical efficiency was set to 80% or higher to recognize a 2-sided significance level of 5%. Sample size was 62 patients (31 per group). Considering a 10% dropout rate, the inclusive sample size was 70 (35 per group).

### Statistical Analysis

SAS version 9.4 (SAS Institute) was used to analyze the study data from June 2021 to April 2022. The Kolmogorov-Smirnov normality analysis was used for examination of the measurement data. If the result indicated normality, it was conveyed as mean (SD). The *t* test was applied for evaluation between the 2 cohorts. If the result did not conform with the median (IQR), the nonparametric Mann-Whitney *U* test was applied. The data of the groups were compared using the χ^2^ test. If the theoretical frequency was too small, Fisher exact probability method was applied, where *P* < .05 was accepted as statistically significant. The primary outcome of HAM-A, secondary outcome of UPDRS, PDQ-39 were assessed by linear mixed model regression test with interaction effects of time and group. Likelihood ratio analysis recommended that simulations with an accidental cutoff had the finest fitting. Effect sizes are described as Cohen *d*.

## Results

### Participants

**Figure 1.**
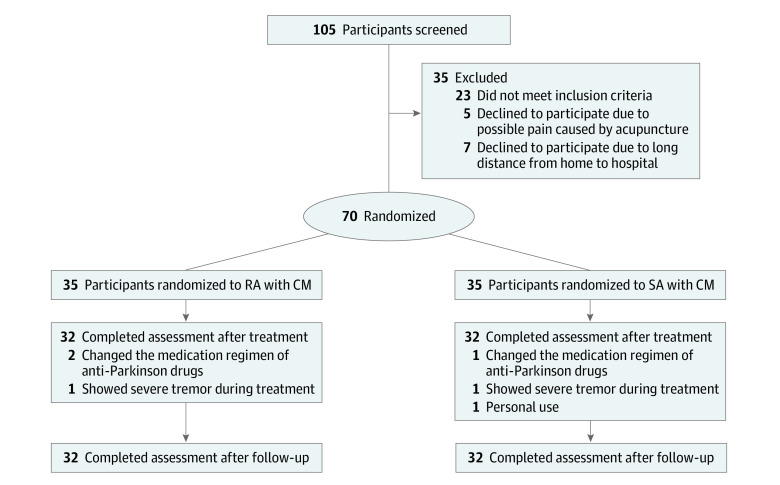
Study Flowchart for Enrollment, Allocation, Follow-up, and Analysis Among randomized patients at Parkinson clinic of the First Affiliated Hospital of Guangzhou University of Traditional Chinese Medicine, 6 patients dropped out before the 12-month follow-up, including 3 who were unable to insist on treatment because of short supply of a certain anti-Parkinson drug in China, 2 who were too nervous during acupuncture, resulting in severe tremor and unable to accept treatment, and 1 who dropped out because of personal reasons.

A total of 105 patients with PD and anxiety were evaluated between June 20, 2021, and February 26, 2022; 70 eligible patients were enrolled, including 34 women and 36 men; 64 patients (91%) completed the intervention and the 8-week follow-up, including 30 women (46.9%) and 34 men (53.1%) with a mean (SD) age of 61.84 (8.47) years (Table 1 and Figure 1).

**Table 1.** Baseline Demographic and Clinical Characteristics of the Included Participants

Characteristic	Total sample (n = 64)	RA (n = 32)	SA (n = 32)
Sex, No. (%)			
Female	30 (46.88)	13 (40.63)	17 (53.13)
Male	34 (53.13)	19 (59.38)	15 (46.88)
Alcohol consumption, No. (%)			
No	52 (81.25)	26 (81.25)	26 (81.25)
Yes	12 (18.75)	6 (18.75)	6 (18.75)
Smoker, No. (%)			
No	47 (73.44)	25 (78.13)	22 (68.75)
Yes	17 (26.56)	7 (21.88)	10 (31.25)
Family history of PD, No. (%)			
No	46 (71.88)	23 (71.88)	23 (71.88)
Yes	18 (28.13)	9 (28.13)	9 (28.13)
Age, mean (SD), y	61.84 (8.47)	61.03 (9.80)	62.66 (6.94)
BMI, mean (SD)	22.23 (1.97)	22.40 (2.41)	22.05 (1.42)
Equivalent daily dose of levodopa, mean (SD), mg	655.52 (242.80)	669.23 (232.90)	641.80 (255.29)
Duration of PD, median (Q1-Q3)^a^, y	5.00 (3.00-8.00)	5.00 (4.00-9.00)	4.00 (2.00-8.00)
UPDRS, mean (SD)	38.05 (14.29)	35.97 (13.53)	40.13 (14.94)
UPDRS I, mean (SD)	5.50 (1.79)	5.16 (2.05)	5.84 (1.44)
HAM-A, mean (SD)	18.81 (3.52)	18.53 (3.61)	19.09 (3.46)
PDQ-39, mean (SD)	58.02 (14.51)	58.94 (19.32)	57.09 (7.30)
PDQ-39-ADL, mean (SD)	10.47 (4.47)	11.09 (5.37)	9.84 (3.31)
PDQ-39-EW, mean (SD)	9.52 (3.92)	9.28 (4.39)	9.75 (3.43)
Blood serum levels of ACTH, mean, M(P25,P75), ng · mL-1^a^	20.99 (20.01,22.19)	20.84 (20.14,22.32)	21.14 (19.89,22.06)
Blood serum levels of CORT, mean, M(P25,P75), pg · mL-1	1126.24 (42.35)	1127.07 (20.20)	1125.41 (22.15)

Abbreviations: ACTH, adrenocorticotropic hormone; ADL, activities of daily living; CORT, cortisol; EW, emotional well-being; HAM-A, Hamilton Anxiety Scale; PD, Parkinson disease; PDQ-39, 39-item Parkinson Disease Questionnaire; RA, real acupuncture; SA, sham acupuncture; UPDRS, Unified Parkinson Disease Rating Scale; UPDRS I, Unified Parkinson Disease Rating Scale I.

^a^ Nonparametric test (Mann-Whitney *U* test) was used.

Six participants (8.5%) abandoned the study. A certain anti Parkinson drug was in short supply in China during the treatment. Thus, some patients had to change the drug during treatment, resulting in withdrawal from the study. The number of patients that dropped out and the reasons for the dropouts are displayed in the CONSORT diagram in Figure 1. Table 1 showed the baseline demographic and clinical characteristics of the included participants.

### Primary Outcome

**Figure 2.**
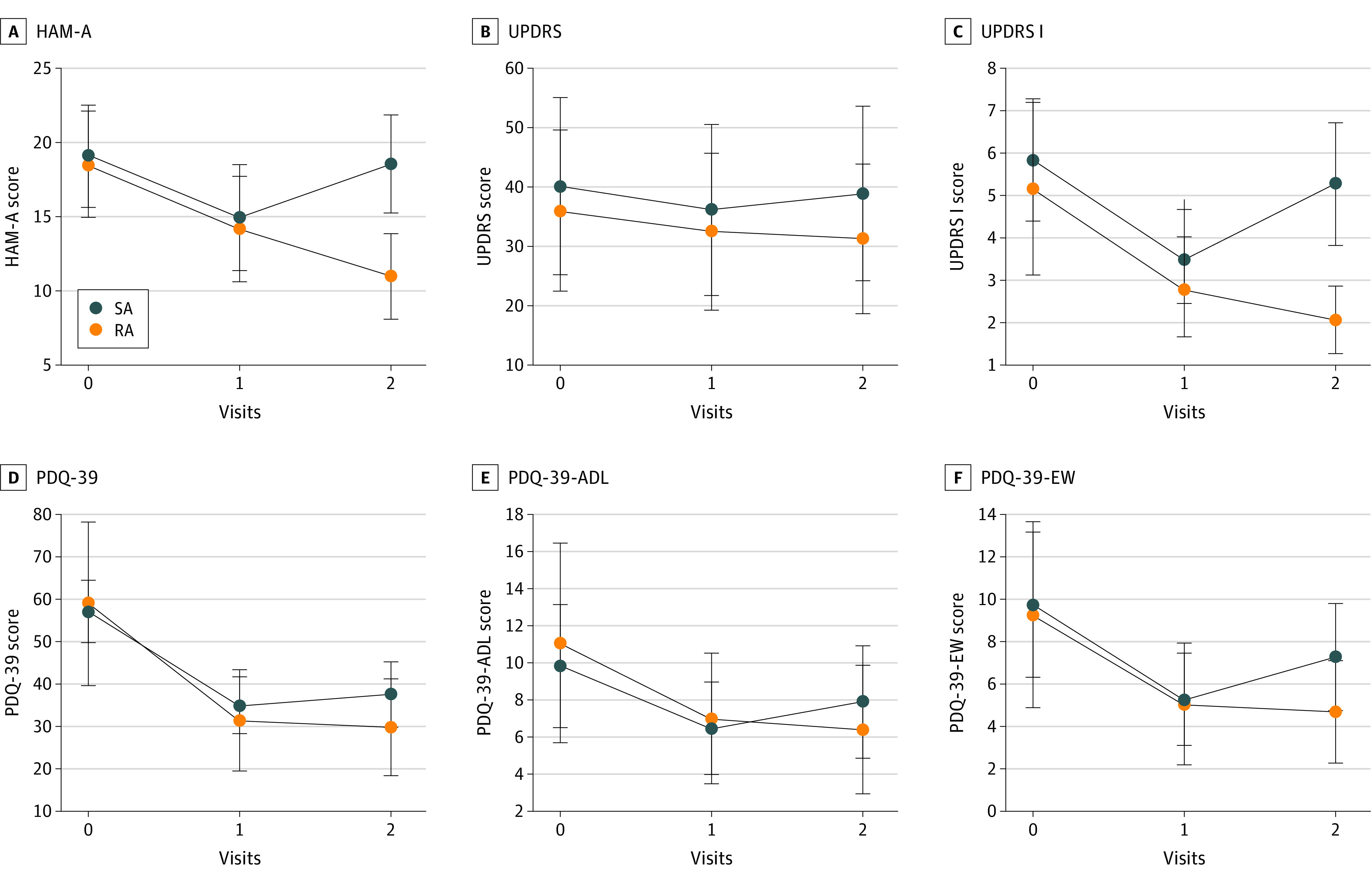
Therapeutic Effects of Acupuncture A, Enhancement level according to HAM-A; B, Improvement in UPDRS score; C, Advancement in UPDRS I score; D, Improvement in PDQ-39 score; E, Enhancement in PDQ-39-ADL score; F, Improvement in PDQ-39-EW score. ADL indicates activities of daily living; EW, emotional well-being; HAM-A, Hamilton Anxiety Scale; PDQ-39, 39-item Parkinson Disease Questionnaire; UPDRS, Unified Parkinson Disease Rating Scale; UPDRS I, Unified Parkinson Disease Rating Scale I.

Table 2 shows comparisons of the primary outcomes of within-groups. Table 3 shows the changes of the main outcome between the 2 groups after treatment. The RA group had a mean reduction of 4.38 ([95% CI, –5.12 to –3.63]; *P* < .001) points in the HAM-A score from baseline. Compared with SA group, patients in the RA group had no significant reduction in HAM-A at the end of treatment (0.22 [95% CI, −0.63 to 1.07]; *P* = .62). After follow-up, patients in the RA group had a significant 7.03-point greater (95% CI, 6.18 to 7.88; *P* < .001) reduction in HAM-A score compared with the SA group. The changes of HAM-A scores between 2 groups are shown in Figure 2.

**Table 2.** Comparison of the Treatment Effects Between 2 Groups and Baseline

Outcome assessments	Mean change from baseline (95% CI)	*P* value	Mean change from baseline (95% CI)	*P* value
	RA group		SA group	
HAM-A				
Posttreatment	–4.38 (–5.12 to –3.63)	<.001	–4.16 (–4.67 to –3.64)	<.001
Follow-up	–7.56 (–8.3 to –6.82)	<.001	–0.53 (–1.05 to –0.02)	.048
UPDRS				
Posttreatment	–3.53 (–4.25 to –2.81)	<.001	–4.03 (–4.82 to –3.24)	<.001
Follow-up	–4.69 (–5.41 to –3.97)	<.001	–1.28 (–2.07 to –0.49)	.002
UPDRS I				
Posttreatment	–2.31 (–2.78 to –1.84)	<.001	–2.28 (–2.59 to –1.97)	<.001
Follow-up	–3.09 (–3.56 to –2.62)	<.001	–0.56 (–0.87 to –0.25)	.001
PDQ-39				
Posttreatment	–27.47 (–32.87 to –22.06)	<.001	–22.03 (–24.18 to –19.88)	<.001
Follow-up	–29.03 (–34.44 to –23.63)	<.001	–19.44 (–21.59 to –17.28)	<.001
PDQ-39-ADL				
Posttreatment	–4.06 (–4.85 to –3.27)	<.001	–3.34 (–3.89 to –2.8)	<.001
Follow-up	–4.66 (–5.45 to –3.87)	<.001	–1.94 (–2.48 to –1.39)	<.001
PDQ-39-EW				
Posttreatment	–4.22 (–4.9 to –3.54)	<.001	–4.47 (–4.94 to –3.99)	<.001
Follow-up	–4.59 (–5.28 to –3.91)	<.001	–2.47 (–2.94 to –1.99)	<.001
ACTH				
Posttreatment	–4.18 (–5.36 to –3.00)	<.001	–1.99 (–3.35 to –6.32)	.005
CORT				
Posttreatment	–19.4 (–63.10 to 24.36)	.37	–12.11 (–60.71 to 36.50)	.62

Abbreviations: ACTH, adrenocorticotropic hormone; ADL, activities of daily living; CORT, cortisol; EW, emotional well-being; HAM-A, Hamilton Anxiety Scale; PDQ-39, 39-item Parkinson Disease Questionnaire; RA, real acupuncture; SA, sham acupuncture; UPDRS, Unified Parkinson Disease Rating Scale; UPDRS I, Unified Parkinson Disease Rating Scale I.

**Table 3.** Comparison of the Treatment Effects in the Real Acupuncture and Sham Acupuncture Groups

Variable	RA (n = 32)	SA (n = 32)	Difference (95% CI)	*P* value^a^	Effect size^b^
Primary outcome					
HAM-A					
Posttreatment	14.16 (3.55)	14.94 (3.59)	0.22 (–0.63 to 1.07)	.62	0.06
Follow-up	10.97 (2.90)	18.56 (3.32)	7.03 (6.18 to 7.88)	<.001	2.06
Secondary outcome					
UPDRS					
Posttreatment	32.44 (13.21)	36.09 (14.37)	–0.50 (–1.55 to 0.55)	.35	−0.12
Follow-up	31.28 (12.59)	38.84 (14.70)	3.40 (2.36 to 4.45)	<.001	0.81
UPDRS I					
Posttreatment	2.84 (1.19)	3.56 (1.11)	0.03 (–0.060 to 0.67)	.92	0.01
Follow-up	2.06 (0.80)	5.28 (1.44)	3.40 (2.36 to 4.45)	<.001	0.98
PDQ-39					
Posttreatment	31.47 (11.94)	35.06 (6.66)	5.44 (–1.46 to 12.33)	.13	0.20
Follow-up	29.91 (11.38)	37.66 (7.61)	9.59 (2.70 to 16.49)	.02	0.35
PDQ-39-ADL					
Posttreatment	7.03 (3.51)	6.50 (2.49)	0.72 (–0.41 to 1.85)	.22	0.16
Follow-up	6.44 (3.45)	7.91 (3.02)	2.72 (1.59 to 3.85)	<.001	0.60
PDQ-39-EW					
Posttreatment	5.06 (2.88)	5.28 (2.17)	–0.25 (–1.22 to 0.72)	.62	−0.06
Follow-up	4.69 (2.42)	7.28 (2.53)	2.13 (1.15 to 3.10)	<.001	0.54
ACTH					
Posttreatment	17.04 (16.42,17.36)	19.39 (17.98,20.18)	2.16 (0.90 to 3.45)	<.001	−1.17
CORT					
Posttreatment	1107.70 (19.29)	1113.31 (22.69)	8.61 (–56.36 to 71.31)	.82	−0.05

Abbreviations: ACTH, adrenocorticotropic hormone; ADL, activities of daily living; CORT, cortisol; EW, emotional well-being; HAM-A, Hamilton Anxiety Scale; PDQ-39, 39-item Parkinson Disease Questionnaire; RA, real acupuncture; SA, sham acupuncture; UPDRS, Unified Parkinson Disease Rating Scale; UPDRS I, Unified Parkinson Disease Rating Scale I.

^a^ Corrected for baseline differences.

^b^ Effect sizes are listed as Cohen *d*; df = 124 for all analyses.

### Secondary Outcomes

UPDRS I and PDQ-39 Emotional Well-being (EW) scores were used as secondary outcomes to reflect the mental states of the participants. At the end of treatment, the variance in enhancement of UPDRS I and PDQ-39-EW between the 2 groups was not statistically significant (UPDRS I: 0.03 [95% CI, −0.06 to 0.67]; *P* = .92; PDQ-39-EW: −0.25 [95% CI, –1.22 to 0.72]; *P* = .62) (Table 3 and Figure 2). After follow-up, the decrease in the UPDRS I score and PDQ-39-EW score of the RA group was significantly greater than the decrease in the score of the SA group (UPDRS I: 3.40 [95% CI, 2.36 to 4.45]; *P* < .001; PDQ-39-EW: 2.13 [95% CI, 1.15 to 3.10]; *P* < .001).

We used UPDRS and PDQ-39 score to assess overall condition and QOL of the participants. At the end of treatment, the variance in reduction of UPDRS and PDQ-39 between the 2 groups was not statistically significant (UPDRS: –0.50 [95% CI, –1.55 to 0.55]; *P* = .35; PDQ-39: 5.44 [95% CI, –1.46 to 12.33]; *P* = .13). At follow-up, the RA group reported a significant reduction in UPDRS score of −3.40 points (95% CI, 2.36 to 4.45; *P* < .001) compared with the SA group. Participants in the RA group had a significant reduction in PDQ-39 score of 9.59 points (95% CI, 2.70 to 16.49; *P* = .02) compared with the SA group (Table 3 and Figure 2). After the acupuncture period, the change of ACTH between the RA and SA groups was statistically significant (2.16 [95% CI, 0.90 to 3.45]; *P* < .001).

### Adverse Events

Four mild adverse reactions occurred during the study. However, no serious adverse events occurred. Adverse reaction records were shown in eFigure 6 in Supplement 1.

## Discussion

To our knowledge, this is the first randomized clinical trial of the effectiveness of an acupuncture treatment regimen targeted for anxiety in patients with PD. There was no significant variance in the degree of improvement between the RA and SA groups after acupuncture. However, we found that 2 months after the treatment, the improvement in the anxiety and mental status of the participants in the RA group was better than that of those in the SA group. In addition to statistical differences, the minimal clinically significant difference (MCID) is principal for interpretation of clinical outcomes.^20^ There is no consensus on MCID of HAM-A. We used anchor-based method to calculate MCID of HAM-A (Supplement 1). Results showed MCID of HAM-A is 4. Therefore, both the RA (65.6%) and the SA groups (62.5%) in the present study reached MCID and showed clinical improvements in anxiety at the end of treatment. At the end of follow-up, 86.8% patients in the RA group and 6.4% patients in the SA group reached MCID.

The clinical improvement in the anxiety of the participants in the RA group was better than the SA group. It can be preliminarily concluded that although there is a certain placebo effect in the short term, acupuncture is clinically effective on anxiety in patients with PD. The placebo effect of acupuncture in the present study disappeared over time; its therapeutic effect was maintained long-term.

There are 2 main reasons for this. First, all the participants of this study are Chinese. Acupuncture, as a traditional Chinese therapy, is highly recognized in China. Thus, participants would generally believe that they have received an effective treatment. Second, as anxiety is a subjective symptom, it is easy to produce a placebo effect during its treatment by using a highly recognized treatment. Thus, to demonstrate the therapeutic effect of acupuncture in terms of mechanism, we used ELISA to measure the serum levels of CORT and ACTH in the 2 groups. CORT and ACTH influence the HPA axis and indirectly reflect a state of anxiety. The serum ACTH levels in the RA group were lower than in the SA group. It may be preliminarily confirmed that acupuncture can reduce the level of ACTH in serum, a finding that is in line with previous results,^21^ where the authors have proved that acupuncture can alleviate increased stress hormone levels and mitigate anxiety.

Overactivation of CORT and ACTH may suppress synthesis and release of the 5-HT and thereby aggravates anxiety in patients with PD.^22,23^ Imbalance of 5-HT is a pivotal factor in the pathogenesis of anxiety in PD.^24^

Therefore, we can infer that acupuncture can reduce 5-HT depletion by inhibiting the hyperactive HPA axis. This hypothesis is consistent with previous studies.^25^ This mechanism may explain the prolonged effect of acupuncture at follow-up. Patients in the SA group may temporarily inhibit the overactivation of HPA axis through the idea of “I have received effective treatment,” but this effect gradually disappears after the treatment.

However, it should be noted that ACTH is an upstream substance of CORT.^26^ The decrease in ACTH level did not cause a decrease of CORT. The intervention duration of this study is short, which may allow for observation of preliminary changes in ACTH level, but not substantial changes in CORT level.

The improvement of the overall condition and QOL of the participants in the RA group was better than that of those in the SA group at follow-up. This may be because reduction of anxiety symptoms leads to reduction of motor symptoms. Improvement of anxiety may also improve the QOL as a whole by promoting the recovery of social roles, and reducing the limitations caused by PD. Thus, it can be preliminarily concluded that acupuncture can improve the overall condition and QOL of patients with PD by alleviating anxiety symptoms. Anxiety, as a tricky nonmotor symptom, is a major challenge in PD treatment.^27,28^ Acupuncture may have a positive effect in the treatment of anxiety in PD.

This study has some strengths. First, this was a double-blind trial conducted through blinding of patients and acupuncture operators. The sham needle devices we developed and used achieve multi-angle needle entry and is highly consistent with the real acupuncture needle. Its good masking effect ensures the validity of the data. Second, this research provides a mechanism basis for the development of acupuncture for patients with PD and anxiety.

### Limitations

This study had some limitations. Regarding evaluation, some items of the HAM-A scale are experienced by PD patients with anxiety and those without anxiety. Thus, there may be some bias in using HAM-A score of at least 14 as the standard for evaluating anxiety in PD. Therefore, more precise criteria are needed to evaluate anxiety in PD. Regarding participants, only Chinese participants were included in this study. Thus, the placebo effect may have been caused by cultural factors. Multicenter research should be considered to reduce influence of cultural differences. Economic benefits and patient acceptance should also be considered in future studies to evaluate the application value of acupuncture.

## Conclusions

This randomized double-blind clinical trial found that acupuncture can effectively ameliorate the anxiety of PD patients. These findings suggest that acupuncture may improve overall motor functions and wellbeing of patients with PD by ameliorating the anxiety.

## Notes

Supplement 1.
Trial Protocol

Supplement 2. **eFigure 1.** The Relationship Between PD and PDA **eFigure 2.** Selection and Location of the Acupoints for Acupuncture **eAppendix.** The Masking Effect of Sham Acupuncture **eFigure 3.** Placebo Acupuncture Appliance Diagram **eFigure 4.** Different Bases of the Appliance **eFigure 5.** Placebo Acupuncture on the Head **eTable 1.** Primary and Secondary Outcomes **eTable 2.** Levodopa Equivalent Dose Conversion **eTable 3.** Operation Rules of Real Acupuncture **eTable 4.** VAS Scores [M (P25, P75)] **eTable 5.** Cohen’s Kappa of Sham Acupuncture
eReferences

Supplement 3.
Data Sharing Statement
