# Supplementary material for: Effectiveness of Acupuncture for Anxiety Among Patients With Parkinson Disease: A Randomized Clinical Trial
Source: JAMA Netw Open. 2022 Sep 21;5(9):e2232133. doi: 10.1001/jamanetworkopen.2022.32133 (PMC9494193; doi:10.1001/jamanetworkopen.2022.32133)
Supplement: Supplement 1. — Trial Protocol [file jamanetwopen-e2232133-s001.pdf]

Protocol of trial

Catalogue

1. General informational 3
1.1 Institution and investigators 3
2. Synopsis 3
3. Background, aim and significance of study 4
3.1 Background 4
3.2 Aims of the Study 6
3.3 significance of study 6
4. Pilot study 7
5. Original protocol 7
Stage 1 7
5.1 Inclusion criteria 7
5.2 Exclusion criteria 7
5.3 Sample size 7
5.4 Randomization 8
5.5 Statistical Analysis 8
5.6 Interventions 8
5.7 quality control 9
5.8 Outcomes 9
Stage 2 10
5.9 Inclusion criteria 10
5.10 Exclusion criteria 10
5.11 Sample size 10
5.12 Standard of suspension 10
5.13 Randomization 10
5.14 Statistical Analysis 10
5.15 Interventions 10
5.16 quality control 10
5.17 Outcomes 11
6.Final protocol of the study 12
6.1 Inclusion criteria 12
6.2 Exclusion criteria 12
6.3 Standard of suspension 12
6.4 Sample size 12
6.5 Randomization 12
6.6 Statistical Analysis 12
6.7 Interventions 13
6.8 quality control 14
6.9 Outcome 14
6.10 Results 15
6.11 Adverse evevnts 16
7 Summary revisions 16

8 The minimal clinically significant difference of calculation........16
9 Discussion........17
10 Ethical Aspects........20
Informed consent for clinical study........20

1. General informational

1.1 Institution and investigators

Institution：Outpatient department of Parkinson's disease, the First Affiliated Hospital of Guangzhou University of traditional Chinese Medicine.

Investigators:

Lixing Zhuang, Professor, Designer and supervisor of acupuncture scheme

Jingqi Fan，PhD, Implementation of experiment，Designer of acupuncture scheme

Nan-bu Wang, PhD, Supervisor of acupuncture scheme

Yu-ting Wang, PhD, Design and manufacture of sham needle

Xin-Liu, PhD, Design and manufacture of sham needle, Designer of acupuncture scheme

Weijing Lu，MM, Implementation of experiment

Ying-jia Li, MM, Implementation of experiment

Yuan-yuan Chen, MM, Implementation of experiment

Keiko Mukaeda, MM, operator assisstants

Hui-Chu, MM, operator assisstants

Wei-qiang Tan, MM, Outcome evaluation

Ming-yue Yan, MM, Outcome evaluation

Li-li Wang, MM, Data statistics and sorting

Xiao-yan Xie, PhD, Data statistics and sorting

2. Synopsis

Title of the study: Effectiveness of Acupuncture for Anxiety among Patients with Parkinson’s Disease: A Randomized Clinical Trial

Study period: 01/06/2021 –15/04/2022

Aim of the study: To investigate the effect of acupuncture versus sham acupuncture for PD patients with anxiety.

Design: Randomized, double-blinded and clinical trial.

Number of patients: N = 70

Inclusion criteria: Patients, diagnosed with idiopathic PD
PD diagnoses from 1 to 4 given according to Hoehn and Yahr scale
Patients’ anxiety assessed following the Hamilton Anxiety Scale (HAM-A) scores ranging from 14 to 29
Patients able to sign informed consent
Patients aged 35–80 years

Outcome measures: Hamilton Anxiety Scale (HAM-A) score
Unified Parkinson’s Disease Rating Scale (UPDRS)
39-item Parkinson Disease Questionnaire (PDQ-39)

Adrenocorticotropic hormone (ACTH) and cortisol (CORT) serum levels

3. Background, aim and significance of study

3.1 Background

3.1.1 PDA poses a significant challenge to the treatment of PD

Although Parkinson's disease (PD) is typically characterized by the presence of rest tremors, bradykinesia, and rigidity, it is also a complex disease with many non-motor manifestations[1]. Parkinson's disease anxiety (PDA) is a non-motor manifestation of Parkinson's disease (PD) that is characterized by a persistent sense of worry, muscle tension, inability to concentrate, headache, and insomnia[2]. PDA is most likely an unintended consequence of PD treatment, etiology, or symptom, which is frequently overlooked and untreated due to its classification as a non-motor symptom. However, approximately 31% of patients with PD are diagnosed with PDA[3], which aggravates the progression of the disease[4,5]and results in symptom fluctuations. Patients with PD and anxiety show greater disability and poorer well-being than these patients without the occurrence of anxiety disturbances[6]. Moreover, disturbances in gait and freezing of gait have been reported to be associated with anxiety symptoms. Thus, anxiety should be regarded as a significant symptom of PD associated with movement disorders[7]. PDA poses a significant challenge to the treatment of PD, endangers patients' lives, and aggravates the motor disorder, imposing a significant financial burden on families and society.

3.1.2 Treatments for PDA are limited

Pharmacology and psychotherapy are the most commonly used treatments for PDA[5,8]. However, no clinical studies have been conducted to confirm the efficacy of these methods[9]. Unwanted side effects, decreased cognitive ability, balance complications, and sedation have all been linked to pharmacological treatments, putting patients at risk of falling[10]. Some anti-Parkinson and anti-anxiety medications cannot be used simultaneously because they may aggravate Parkinson's disease[11]. Antidepressant drugs, such as serotonin reuptake inhibitors (SSRI), combined with anti-Parkinson drugs, such as monoamine oxidase B inhibitors (MAO-BI), can easily aggravate tremor symptoms[12] Psychotherapy such as cognitive behavioral therapy has few side effects but takes a long time and is costly for patients[13]. There is a need for more appropriate complementary therapies for the treatment of PDA.

3.1.3 Acupuncture shows good effect on anxiety

Patients with anxiety benefit from more effffective and safer forms of complementary alternative interventions, such as acupuncture[14].

A guideline recommends non drug therapy for patients with mild to moderate anxiety[15]. Acupuncture is one of the most popular complementary and alternative medicine treatment methods due to its short duration, minimal side effects, low cost, high acceptance, and significant curative effect[10]. Acupuncture is one of the non-pharmacological techniques, which can be used for treating anxiety[16].The positive effect of acupuncture on anxiety has been verified in many studies[17]. Most of the studies suggested that the acupuncture group was more effective than traditional medicine alone and sham acupuncture in the treatment of anxiety.

3.1.4 Acupuncture shows good effect on Parkinson's disease

Under the guidance of tradition Chinese theory and mechanism research, acupuncture is applied to the clinical research of PD. Increasing clinical studies have confirmed that acupuncture can significantly improve motor and non-motor symptoms (NMS) of PD[18,19].

Li et al.[19] randomly divided patients with PD into sham-acupuncture, waiting, and acupuncture groups, and significant improvement was observed in the UPDRS in the acupuncture group, especially in their tremors scores. To observe bradykinesia, Fukuda et al. compared the effectiveness of acupuncture in different kinds of gait disorders. Results demonstrated that step length, gait speed, and floor reaction force were all improved[20]. For rigidity, Toosizadeh compared EA and sham treatment to treat patients with PD and found that there was a significant reduction ($p < 0.02$) in the EA group in UPDRS fall status (67%) and rigidity (48%)[21]. A review concluded that it is possible that acupuncture can resolve motor symptoms by protecting dopaminergic neurons from toxic insults and increasing dopamine production in the brain, inducing the release of neurotrophic factors, enhancing antioxidant agents, and inhibiting inflammatory responses[22]. This supports the point that acupuncture relieves motor symptoms in PD by reinforcing the deficiency. Nevertheless, we couldn't find data to confirm acupuncture's utility in curing patients with PDA.

<u>3.1.5 Whether the effective mechanism of acupuncture is related to the effect of placebo?</u>

"Acupuncture and moxibustion of traditional Chinese medicine" was officially listed as the "representative list of human intangible cultural heritage" in 2010, and is widely used worldwide. Despite over 3500 clinical studies on acupuncture, there is still debate over a true effect.In explaining whether acupuncture has placebo effect, it is important to go further by using comfort acupuncture[23]. Since 1999, the article on sham acupuncture has been cited by the Science Citation Index (SCI) for the first time.Sham acupuncture is one of the research hotspots in the field of acupuncture. Compared with other comfort control methods, such as control with adjacent false points, control with non disease related true points, control with shallow needling of false points, and control with false electricity, sham acupuncture devices are more widely used in research[24]. Therefore, it is the key to confirm the curative effect of acupuncture to select a suitable false acupuncture device for a randomized controlled double-blind trial.

At present, the comfort acupuncture device used in clinical trials and research has the following shortcomings: the needle entry angle is single, which can not be applied to the acupoints requiring flat or oblique ; it does not have the conditions to apply lifting, inserting, twisting and other techniques, and the feeling under the needle is quite different from the real acupuncture, so it is impossible to blind the performer; the fixation of the device is poor, especially in the parts with rich hair, it is easy to slip, etc[25].

In order to meet the experimental needs, our team desigened and made a sham needle device. It can realize multi angle needle feeding, with good fixation, and can blind patients and operators at the same time. This device is an auxiliary device with reasonable design for placebo acupuncture research, so as to make double-blind placebo acupuncture control possible. This new auxiliary acupuncture device includes an acupuncture device and a placebo acupuncture device with exactly the same appearance. Both of them are composed of a hemispherical base and a telescopic tube. Through the rotation of the telescopic tube in the notch of the base, the insertion angle of needles can be adjusted from 15 degrees to 165 degrees. The operation of twisting and lifting and inserting can be carried out through the horizontal rotation and vertical sliding of the telescopic tube. A silicone needle pad is arranged in the base, which can simulate the blocking feeling of skin and muscle during needle insertion. The bottom

of the base is attached with hydrogel, which has good fixity. The auxiliary device is applicable to multiple parts of the human body and can effectively reduce the risk of unblinding.

This study explored whether placebo effect of acupuncture exits through self-made comfort needle device.

3.2 Aims of the Study

3.2.1 Primary objective:

To investigate the effect of acupuncture versus sham acupuncture for PD patients with anxiety.

3.2.2 Secondary objective:

To investigate whether acupuncture could improve the well being of PD patients by resolving anxiety.

To explore the possible mechanism of acupuncture in the treatment of PDA by assaying serum CORT and ACTH.

3.3 significance of study

To our knowledge, this is the first randomized controlled trial of the effectiveness of an acupuncture treatment regimen targeted for PDA.

This was a double-blind trial conducted through blinding of patients and evaluators. We administered acupuncture at a 15° angle and a 90° angle. The current sham needle could not be used to achieve this multi-angle needle entry. The sham needle we developed and used in this study was highly consistent with the real acupuncture needle in terms of shape, angle, and feel. Its masking effect was good, which can ensure the validity of the data. the masking effect of sham acupuncture was shown in supplement

The multi angle sham acupuncture needle set provides a technical reference for acupuncture research in the future.

This research provides a basis for the development of effective alternative therapies for PDA.

4. Pilot study

A pilot case investigation was performed to refine the methodology of acupuncture to generate the data to calculate the sample size for the randomized controlled trial.

We retrospectively selected 6 patients who received acupuncture treatment in Parkinson's clinic. Another 14 patients were recruited to receive sham acupuncture treatment or real acupuncture treatment.There were 10 cases in each group.

The mean HAM-A score of patients who received acupuncture combined with anti-Parkinson drugs was 15.3, standard deviation was 2.55, whereas that of patients who received the sham acupuncture with anti-Parkinson drugs was 13.2, standard deviation was3.18.

5. Original protocol

Stage 1

5.1 Inclusion criteria

- Idiopathic PD diagnosis
- Hoehn and Yahr staging scale stages 1 to 4
- HAMA scale score range 14 to 29
- Ability to provide written informed consent
- Men or women aged 35 to 80 years

5.2 Exclusion criteria

- Significant neurologic, renal, cardiovascular, or hepatic impairment
- Any disease that can result in Parkinson’s syndrome or other conditions that are suspected to be related to the patient’s symptoms
- Significant cognitive impairment as defined by the score of the Montreal Cognitive Assessment (MOCA) of <23
- No significant response to high-dose levodopa therapy
- Medical or psychological condition that makes participation in the study challenging
- Use or dependence on drugs or alcohol that could affect participation in the study
- Within 30 days of treatment initiation, exposure to the study drug or acupuncture
- Allergy or intolerance to acupuncture
- Inability to adhere to the study protocol as determined by the investigator.

5.3 Sample size

The sample size was calculated using the primary outcome, which is a shift in the HAMA score from baseline. According to preliminary results,The mean HAM-A score of patients who received acupuncture combined with anti-Parkinson drugs was 15.3, standard deviation was 2.55, whereas that of patients who received the sham acupuncture with anti-Parkinson drugs was 13.2, standard deviation was 3.18.

we determined that a sample comprising 64 patients (32 in each group) would have a power of 80% or more to identify a two-sided significance level of 5%. The overall sample size needed for the study is 70 (35 in each group), anticipating a 10% dropout rate. If the patient cannot adhere to a course of acupuncture, his condition suddenly worsens during the treatment process, or the treatment plan of anti-Parkinson drugs needs to be changed, the acupuncture regimen will be stopped.

5.4 Randomization

Participants will be allocated to two intervention groups at random in a ratio of 1:1 to receive acupuncture plus anti-Parkinson drugs or anti-Parkinson drugs with sham acupuncture. A randomization sequence will be generated by a statistician not participating in this trial using SPSS Statistics 26.0. The assignment sequences will be concealed in sequentially numbered, sealed, and opaque envelopes to ensure sequence confidentiality.

5.5 Statistical Analysis

To ensure the integrity and accuracy of data, two statisticians will independently perform statistical analyses using Statistics 26.0 (IBM SPSS Statistics Inc., Chicago, USA), establish the database, and provide proofreading for logic. Enumeration data will be expressed as percentages, whereas measurement data will be expressed as mean ± standard deviation. A normality test will be performed for the measurement data. For variables that are determined to be regularly distributed, a t-test will be employed, and for those that are not, a rank-sum test will be utilized. The rank-sum test and nonparametric test will be employed for graded data, and the chi-square test will be applied to counted data. Repeated-measures analysis of covariance (ANCOVA) will be used for the UPDRS, Hoehn–Yahr, HAMA, and SF-36 scales. The rank-sum test will be applied to compare efficacy. Serological index values will be analyzed using covariance. Two-sided tests will be used for all hypothesis testing, with $P < 0.05$ as the standard of statistical significance.

5.6 Interventions

5.6.1 Acupuncture Group.

Participants assigned to the acupuncture group will receive twelve 30 min sessions of acupuncture (once every other day, four times a week for four weeks) with clinical monitoring only (CMO) and maintain their original dosage of anti-Parkinson drugs. If the dosages of GV 21, GV 19, and 1.5 cun next to GV 20 bilaterally, with the width of the patient's thumb joint being taken as one cun). The acupoints' positions are described by the National Standard of the People's Republic of China's (GB/T 12346–2006) standard for the name and location of the acupoints, which was established in 2006. Acupuncture will be performed with disposable, sanitized stainless steel needles (Tianxie, Suzhou Medical Appliance Factory, Suzhou, China; 0.25 × 25 mm, 0.25 × 40 mm). GV 24 (*shen ting*), GV 29 (*yin tang*), and *Si Shen Zhen* adopt 0.25 × 25 mm acupuncture needles with a 45-degree angle. Bilateral HT7 (*shen men*) and bilateral SP 6 (*san yin jiao*) will use 0.25 × 40 mm acupuncture needles with a 90-degree angle. After routine disinfection, the acupuncturist inserts the needles into the corresponding acupoints and stimulates all the needles by lifting, twirling, and thrusting to obtain the *de qi* sensation (a combination of feelings, such as numbness, soreness, and heaviness). CMO will involve providing patients with general educational leaflflets on dealing with anxiety that were retrieved from web portals of the French and Netherlands psychiatric associations.

5.6.2 Control Group. Participants assigned to the control group will receive twelve 30 min sessions of acupuncture with sham acupuncture needles (once every other day, four times a week for four weeks) with CMO and maintain the original dosage of their anti-Parkinson drugs. If dosages need to be increased or decreased during the trial to relieve symptoms, the change will be recorded on the case report form. For GV 24 (*shen ting*), GV 29 (*yin tang*), and *Si Shen Zhen,* 0.25 × 25 mm acupuncture needles with a 45-degree angle will be used. For Bilateral HT7 (*shen men*) and bilateral SP 6 (*san yin jiao*), 0.25 × 40 mm acupuncture needles with a 90-degree angle will be used. In this study, some acupoints need to be penetrated at an angle of 15°. 1is acupuncture tool consists of a base sticking to the skin and a sleeve. In particular, there are two types of bases in this acupuncture tool, one

is hollow while the other is sealed by the adhesive layer. Specifically, we will paste this special acupuncture tool on the skin first. In the acupuncture group, we will put acupuncture needles through the cannula and pierce them into the skin through the perforated base to achieve the acupuncture effect. However, when used in the control group, a special placebo needle will be used to pass through the cannula and pressed against the adhesive layer and the skin, and a slight pressure will be applied to ensure a placebo effect similar to acupuncture. In this way, we hope to achieve a single-blind effect on the patients to verify the placebo effect in acupuncture.

5.7 quality control

A research workbook will be established. All the investigators will be trained and tested for conformance with preset standards before the beginning of each stage. Each participant will have a fixed physician and time points for treatment to ensure consistency in the intervention effect. Since the use of anti-Parkinson drugs will affect the physician's evaluation of patient symptoms, the participants will be evaluated for PD symptoms 4 hours after taking their medication. All acupuncturists participating in this project have received formal education at Traditional Chinese Medicine universities, obtained physician qualification certificates, and possess more than 3 years of clinical experience in acupuncture.

5.8 Outcomes

Primary and secondary outcomes

Domain	Instrument
Anxiety state	HAMA
Physical Status	Hoehn–Yahr Rating Scale
Clinical assessment	UPDRS
Blood serum levels of ACTH	ELISA Kit of ACTH
Blood serum levels of CORT	ELISA Kit of CORT
Blood serum levels of CRF	ELISA Kit of CRF
Blood serum levels of 5-HT	ELISA Kit of 5-HT

HAMA, Hamilton Anxiety Scale; UPDRS, Unified Parkinson's Disease Rating; ACTH, adrenocorticotropic hormone; CORT, cortisol; CRF, corticotropin-releasing factor; 5-HT, serotonin.

Stage 2

5.9 Inclusion criteria

The inclusion criteria for stage 2 are the same as for stage 1.

5.10 Exclusion criteria

The exclusion criteria for stage 2 are the same as for stage 1.

5.11 Sample size

The sample size was calculated using the primary outcome, which is the shift in HAMA score from baseline. According to preliminary results, the mean HAMA score in patients who received acupuncture combined with anti-Parkinson drugs was 15.8, while it was 13.9 in patients who received anti-Parkinson drugs alone. A sample size comprising 74 patients (37 in each group) would have a power of 80% or more to identify a two-sided significance level of 5%. The overall sample size needed for the study is 82 (41 in each group), anticipating a 10% dropout rate. If a patient cannot adhere to a course of acupuncture, his condition suddenly worsens during the treatment process, and the treatment plan of anti-Parkinson drugs needs to be changed, the treatment will be stopped.

5.12 Standard of suspension

1. Condition of participants suddenly worsens during the treatment process
2. Participants cannot adhere to a course of acupuncture
3. The change of dosage of anti Parkinson drugs leads to the change of equivalent dose of levodopa.

5.13 Randomization

Patients will be randomly allocated in a 1:1 ratio into two intervention groups to receive acupuncture in combination with anti-Parkinson drugs or anti-Parkinson drugs alone. A randomization sequence will be generated by a statistician not participating in this trial, using SPSS Statistics 26.0 (IBM SPSS Statistics Inc., Chicago, USA). The assignment sequences will be concealed in sequentially numbered, sealed, opaque envelopes to ensure sequence confidentiality.

5.14 Statistical Analysis

All data will be analyzed using intention-to-treat (ITT) analysis and the per-protocol set (PPS) for coherence. As in stage one, normality tests will be performed on the measurement data. A t-test will be performed for variables found to be normally distributed, and the rank-sum test will be used for those not normally distributed. The rank-sum test and nonparametric test will be used for the graded data, and the chi-square test will be applied to counted data. Repeated-measurements ANOVA will be used for the UPDRS, Hoehn–Yahr, HAMA, and SF-36 scales. The rank-sum test will be used to compare efficacy.

5.15 Interventions

5.15.1 Acupuncture Group. Interventions for the acupuncture group in stage 2 will be the same as those in stage 1.

5.15.2 Control Group. Participants in the control group will be required to obtain CMO and maintain the original dosage of their anti-Parkinson drugs. If the dosages need to be increased or decreased during the trial to relieve symptoms, the change will be recorded in the case report form.

5.16 quality control

A research workbook will be established. All the investigators will be trained and tested for conformance with preset standards before the beginning of each stage. Each participant will

have a fixed physician and time points for treatment to ensure consistency in the intervention effect. Since the use of anti-Parkinson drugs will affect the physician's evaluation of patient symptoms, the participants will be evaluated for PD symptoms 4 hours after taking their medication. All acupuncturists participating in this project have received formal education at Traditional Chinese Medicine universities, obtained physician qualification certificates, and possess more than 3 years of clinical experience in acupuncture.

5.17 Outcomes

Primary and secondary outcomes

Domain	Instrument
Anxiety state	HAMA
Physical Status	Hoehn–Yahr Rating Scale
Clinical assessment	UPDRS
Blood serum levels of ACTH	ELISA Kit of ACTH
Blood serum levels of CORT	ELISA Kit of CORT
Blood serum levels of CRF	ELISA Kit of CRF
Blood serum levels of 5-HT	ELISA Kit of 5-HT

HAMA, Hamilton Anxiety Scale; UPDRS, Unified Parkinson's Disease Rating; ACTH, adrenocorticotropic hormone; CORT, cortisol; CRF, corticotropin-releasing factor; 5-HT, serotonin.

6.Final protocol of the study

6.1 Inclusion criteria

- patients, diagnosed with idiopathic PD14;
- PD diagnoses from 1 to 4 given according to Hoehn and Yahr scale;
- patients' anxiety assessed following the Hamilton Anxiety Scale (HAM-A) scores ranging from 14 to 29;
- patients able to sign informed consent;
- patients aged 35–80 years.

6.2 Exclusion criteria

- major cognitive impairment diagnosed following the Montreal Cognitive Assessment score, less than 23;
- irresponsiveness to treatment with high doses of levodopa;
- drugs or alcohol abuse;
- revevied acupuncture therapy within 30 days before treatment;
- took anti anxiety drugs within 30 days before treatment;
- major neurologic, renal, cardiovascular, or hepatic deficiency;
- intolerance to acupuncture.
- Dropout criteria: 1) If participants' anxiety symptoms worsen or even cannot be controlled during the treatment, it is recommended that the patient need to take anti anxiety drugs after evaluation by a professional psychologist. 2. Due to the aggravation of the patient's condition during the treatment, it is recommended to change anti Parkinson medications after the evaluation of professional neurologists.

6.3 Standard of suspension

1. Condition of participants suddenly worsens during the treatment process
2. Participants cannot adhere to a course of acupuncture
3. The change of dosage of anti Parkinson drugs leads to the change of equivalent dose of levodopa.

6.4 Sample size

The sample size was determined by the variation in the HAM-A score in pilot study.The mean HAM-A score of patients who received acupuncture combined with anti-Parkinson drugs was 15.3±2.55, whereas that of patients who received the sham acupuncture with anti-Parkinson drugs was 13.2±3.18. The power of statistical efficiency was set to 80% or higher to recognize a two-sided significance level of 5%, which was 62 patients (31 per group). Considering a 10% dropout rate, the inclusive sample size was 70 (35 per group).

6.5 Randomization

The participants were randomly allocated into the RA and SA groups in a ratio of 1:1. Randomization and biding was created by a mathematician, who was not involved in the study, by SPSS Statistics 26.0 (IBM SPSS Statistics Inc., Chicago, USA).

6.6 Statistical Analysis

SAS 9.4 (Cary, NC) was used to analyze the study data. The Kolmogorov–Smirnov normality

analysis was used for examination of the measurement data. If the result indicated normality, it was conveyed as mean ± standard deviation. The t-test was applied for evaluation between the two cohorts. If the result did not conform with the median (q1-q3), the nonparametric Mann –Whitney U test was applied. The data of the groups were compared using the x^2 test. If the theoretical frequency was too small, Fisher's exact probability method was applied, where $P < 0.05$ was accepted as statistically significant. The primary outcome of HAM-A, secondary outcome of UPDRS, PDQ-39 were assessed by linear mixed model regression test with interaction effects of time and group. Likelihood ratio analysis recommended that simulations with a accidental cut off had the finest fitting. Effect sizes are described as Cohen's d.

6.7 Interventions

Participants in RA and SA groups received 30 min acupuncture once per day, three times per week for a period of 8 weeks, with fixed prescriptions according to the traditional Chinese medicine theory and the information in previous articles on PD and anxiety.[26] All participants received acupuncture at GV 24 (shen ting), GV 29 (yin tang), bilateral HT7 (shen men), bilateral SP 6 (san yin jiao), and Si Shen Zhen, which included four acupoints, including GV 21, GV 19, and 1.5 cun next to GV 20 bilaterally. The names and locations of acupoints are labelled following the National Standard of the People's Republic of China (GB/T 12346–2006), established in 2006.

Real acupuncture: After disinfecting the skin, the participants were laid flat on the treatment bed and the acupoint sites were exposed. Operation assistants fixes the acupuncture auxiliary device on the acupoint skin; Secondly, acupuncture operators quickly tap the top of the inner tube with the index finger, so that the inner tube drives the acupuncture needle to pierce downward. Real needle tip pierces the human skin. After the treatment, the acupuncture auxiliary device can be removed by a operation assistants. In the process of acupuncture, neither the acupuncture operator nor the patient can know their own grouping. Patients wear eye masks throughout the whole process to avoid breaking the blind. Acupuncture was performed using one-use, sterile, stainless steel needles (Tianxie, Suzhou Medical Appliance Factory, Suzhou, China; 0.25 × 25 mm, 0.25 × 40 mm). Acupuncture operation process and acupoints was shown in eFigure 3. Acupuncture was applied at GV 24 (*shen ting*), GV 29 (*yin tang*), and *Si Shen Zhen* using a 0.25 × 25 mm needle inserted at 45 angle, the needle depth is 15-20mm. Acupuncture was applied at bilateral HT7 (*shen men*) and bilateral SP 6 (*san yin -jiao*) using a 0.25 × 40 mm needle inserted at a 90˚ position.

The needle depth of HT7 (*shen men*)is 15-20mm, the needle depth of SP 6 (*san yin -jiao*) is 25-30mm. After needle insertion, twist the needle for 1 minute at a frequency of 180 ~ 200 rpm to achieve *de qi.*

Sham acupuncture: Participants assigned to the control group will receive twelve 30 min sessions of acupuncture with sham acupuncture needles (three times a week for eight weeks) with CMO and maintain the original dosage of their anti-Parkinson drugs. For GV 24 (*shen ting*), GV 29 (*yin tang*), and *Si Shen Zhen*, 0.25 × 25 mm acupuncture needles with a 45-degree angle will be used. For Bilateral HT7 (*shen men*) and bilateral SP 6 (*san yin jiao*), 0.25 × 40 mm acupuncture needles with a 90-degree angle will be used. In this study, some acupoints need to be penetrated at an angle of 15°. This is acupuncture tool consists of a base sticking to the skin and a tube. In particular, there are two types of bases in this acupuncture tool, one is hollow while the other is sealed by the adhesive layer. Specififically, we will paste this special acupuncture tool on the skin first. In the real acupuncture group, we will put acupuncture needles through the tube and pierce them into the skin through the perforated base to achieve the acupuncture effect. However, when used in the control group, a special placebo needle will be used to pass through the tube and pressed against the

adhesive layer and the skin, and a slight pressure will be applied to ensure a placebo effect similar to acupuncture. In this way, we hope to achieve a single-blind effect on the patients to verify the placebo effect in acupuncture.Supplement 2 show the structure of the sham acupuncture needle.

After disinfecting the skin, the participants were laid flat on the treatment bed and the acupoint sites were exposed. Operation assistants fixes the acupuncture auxiliary device on the acupoint skin; Secondly, acupuncture operators quickly tap the top of the inner tube with the index finger, so that the inner tube drives the acupuncture needle to pierce downward. Sham needle tip pierces the human skin. After the treatment, the acupuncture auxiliary device can be removed by a operation assistants. In the process of acupuncture, neither the acupuncture operator nor the patient can know their own grouping. Patients wear eye masks throughout the whole process to avoid breaking the blind.

Acupuncture was performed using special disposable, sanitized, sham stainless steel needles. The participants in the SA group underwent a non-insertion procedure applied at the same acupoints and using the method as in the RA group. After needle insertion, twist the needle for 1 minute at a frequency of 180 ~ 200 rpm. The needle was kept for 30 minutes in both groups, and then the device was removed at end of the treatment.

6.8 quality control

All patients in RA and SA groups received CM and maintained the original doses of their anti-Parkinson medications. If the drugs needed to be changed, the observer recorded and calculated whether the equivalent dose of levodopa changed as well. Observers and operators who maintained daily contact with participants and recorded any symptoms that may reflect changes in the patient's condition were included for analysis.

6.9 Outcome

Primary outcome

The primary outcome was the Hamilton Anxiety Scale (HAM-A score),which apply for assessing the degree of anxiety. It consists of 14 symptomatic definition elements, with a total possible score of 56.A total score of 7 to 56 indicates anxiety.

Secondary outcomes

The unified Parkinson Disease Rating Scale (UPDRS) and the 39-item Parkinson Disease Questionnaire (PDQ-39) as well as the blood serum levels of ACTH (adrenocorticotropic hormone) and CORT (cortisol) were the secondary outcomes. The serum

levels of CORT and ACTH influence the activation state of the HPA axis, which reflects the state of anxiety.19 The serum CORT and ACTH levels of the participants were measured four hours after medications were taken on the day before and the day after treatment. For analysis, 4 mL of blood was extracted from the elbow vein and centrifuged for 10 min at 12000 RPM in a Boke high-speed freezing centrifuge. A 2 mL supernatant was obtained and stored in a refrigerator at - 80 ˚C. Serum CORT and ACTH levels were evaluated by enzyme linked immunosorbent assay (ELISA) . The ELISA kits were purchased from Jiangsu enzyme immunoassay Industry Co., Ltd. (batch numbers of CORT, mm-0027h2; ACTH, MM-1449H2). A Multiskan FC microplate reader was used to measure the absorbance value of each hole at a wavelength of 450 nm, and the corresponding sample content was

calculated according to the linear regression equation of the standard curve.

6.10 Results

Participants

A total of 105 patients with PDA were evaluated between June 20, 2021, and February 26, 2022, and 70 eligible patients were enrolled, including 34 women and 36 men. 64 patients (91%) completed the intervention and the two-month follow-up,including 30 women(46.9%) and 34 men(53.1%) with a mean (SD) age of 61.84(8.47) years.

Six of them (8.5%) abandoned the study. A certain anti Parkinson's drug was in short supply in China during the treatment. Thus, some patients had to change the drug during treatment, resulting in withdrawal from the study. The number of patients that dropped out and the reasons for the dropouts are displayed in the CONSORT diagram in Figure 1.

Primary outcome

Table 3 shows comparison of the primary outcomes of within- groups .Table 4 shows the changes of the main outcome between the two groups after treatment.RA group had a mean reduction of 4.38[95% CI-5.12 to -3.63]points in the HAM-A score from baseline. Compared with SA group, patients in RA group had no significantly reduction in HAM-A at the end of treatment (0.22 [95% CI, −0.63 to 1.07]).After follow-up, patients in RA group had significantly reduction in HAM-A score of 7.03 compared with SA group(95% CI, 6.18 to 7.88; P<0.001).

Secondary outcomes

UPDRSI and PDQ-39 Emotional Well-being (EW) scores were used as secondary outcomes to reflect the mental states of the participants. At the end of treatment, the variance in enhancement of UPDRSI and PDQ-39EW between the two groups was not statistically solid (0.03[95% CI, −0.06 to 0.67]；P=0.92;-0.25(-1.22 to 0.72);P=0.62, respectively) (Table 3 and Fig. 2c,f). After follow-up, the decrease in the UPDRS I score and PDQ-39 EW score of the RA group was significantly greater than the decrease in the score of the SA group(3.40[95% CI, 2.36 to 4.45];2.13[95% CI,1.15 to 3.10],respectively)(P<0.001 for all).

We evaluated the motor and non-motor indications of the patients by UPDRS score to determine whether acupuncture improved the overall condition of the patients by improving anxiety. PDQ-39 score was used as a secondary outcome to assess the QOL of the participants.At the end of treatment, the variance in reduction of UPDRS and PDQ-39 between the two groups was not statistically significant (-0.50[95% CI,-1.55 to 0.55];P=0.35; 5.44[95% CI,-1.46 to 12.33];P=0.13) (Table 3 and Fig. 2b,d). At follow-up, RA group reported a significant reduction in UPDRS score of −3.40 points (95% CI,2.36 to 4.45; P < .001) compared with the SA group. Besides, participants in RA group had a significant reduction in PDQ-39 score of 9.59 points (95% CI,2.70 to 16.49 ; P =0 .02) compared with the SA group.

After the acupuncture period, the within-group improvement in the serum CORT level of participants in the RA and SA groups was not statistically substantial(-19.4[95% CI,-63.10 to 24.36],P=0.37;-12.11[95% CI,-60.71 to 36.50)],P=0.62,respevtively) . In addition, the variance between the groups was not statistically significant (8.61[95% CI,-56.36 to 71.31],P=0.82;). The within-group improvement in the serum ACTH level of the participants in the RA group was statistically significant (-4.18[95% CI,-5.36 to -3.00],P<0.001)..Besides, the change between the RA and SA groups was statistically significant.(2.16[95% CI,0.90 to3.45],P<0.001).

6.11 Adverse evevnts

Of the adverse events recorded, two were cases of more bleeding after needle injection and appearance of a slight hematoma under the skin. The size of the hematoma was about 1cm × 1cm. Pressure was applied for 5–10 minutes to stop the bleeding and the area was pressed with a cotton ball soaked with 95% alcohol to reduce the swelling. The hematoma was absorbed after 2–3 days. The other two cases were cases of difficulty removing the acupuncture needle due to mental tension and muscle spasm. When this happened, the muscles around the acupoint were tapped and massaged and the needle was released smoothly.

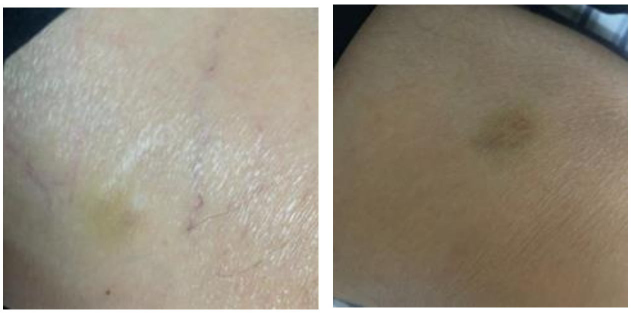

Hematoma appeared after acupuncture.

7 Summary revisions

Compared with the original plan, we have reduced the number of acupuncture from four times a week to three times a week, in order to avoid the traffic burden caused by commuting to and from the hospital every day. We used PDQ-39 instead of SF-36 to evaluate the patient's ability to live scores. Because compared with SF-36, PDQ-39 is more targeted in assessing the quality of life of patients with Parkinson's disease.Due to the problem of reagent transportation, we finally chose ACTH and CORT to test the serum content. In the original plan, we designed a two-stage study. In the first stage, in order to eliminate the placebo effect caused by acupuncture, we used sham acupuncture as control. In the second stage, blank control was used to further confirm the clinical effect of acupuncture. In this study, although real acupuncture shows better therapeutic effect, sham acupuncture and real acupuncture also have certain acupuncture effect, so the second stage has not been implemented.

In the previous design, we only considered the statistical significance of acupuncture in the treatment of PDA, ignoring the clinical significance. Statistical significance does not fully represent clinical significance.Therefore, in the actual study, we used the anchoring method to calculate the minimum clinically important difference, and compared it with the treatment results to prove whether acupuncture has clinical significance in alleviating PDA.

8 The minimal clinically significant difference of calculation

Deciding on the smallest change in an outcome that constitutes a clinically meaningful treatment effect (the minimum clinically important difference [MCID]) is fundamental to interpreting clinical trial outcomes, making clinical decisions, and designing studies with sufficient statistical power to detect any such effect. In addition to statistical differences, whether a treatment method has clinical significance is more paramount for patients.There is no consensus on MCID for outcomes in HAM-A.

At present, there is no unified method for calculating MCID. The main methods are anchor based method, distribution method, consensus method, literature method, etc.

The anchor based method method estimates the target measurement by associating an independent measurement index, namely, the external benchmarking MCID of the indicator.

The anchor based method is used to calculate MCID of HAM-A , and UPDRS is used as the main anchor. Pearson correlation analysis was used to calculate the correlation coefficient of 0.251. The difference is skewed, with a median of 4.

(1) anchoring method (effective standard method)

Firstly, select an appropriate anchor and report the correlation coefficient between the anchor and the test scale. Secondly, calculate the questionnaire score difference of patients who differ by at least one grade in anchor options before and after the intervention. If the difference obeys the normal distribution, the mean value of the difference is taken as the MCID. If the difference follows a skew distribution, the median is MCID.

Therefore, after preliminary calculation, the MCID of HAM-A is 4, that is, if the difference of HAM-A before and after treatment is greater than 4, it can be preliminarily confirmed that acupuncture has clinical significance for PDA.

Therefore, Both the RA （65.6%）and the SA groups（62.5%）in the present study reached MCID and showed clinically improvements in anxiety at the end of treatment.At the end of follow-up , 86.8% patients in RA group and 6.4% patients in SA group reached MCID.

The clinically improvement in the anxiety of the participants in the RA group was better than SA group .

9 Discussion

Anxiety is more common in PD patients than in the general population[27]. PDA may be related to L-DOPA treatment, dopamine deficiency etiology, and complex PD symptoms[28].

The symptoms of PD are complex. In addition to motor symptoms, there are a series of non-motor symptoms, such as sleep disorder, constipation, depression, etc. These complex symptoms seriously affect the quality of life of patients, resulting in emotional burden and provoking the development of disorders[27]. Deficiency of dopamine in PD results in increased firing rate of the locus coeruleus(LC), which suppresses the synthesis and release of the 5-HT neurons and thereby aggravates the anxiety levels in patients with PD. However, excessive dopamine intake can easily affect the balance between dopamine and 5-HT, resulting in PDA[29]. The decrease of endogenous dopamine in the striatum of PD patients and the excessive intake of dopamine during long-term drug use easily cause the occurrence of PDA. PDA may be an adverse outcome of disease progression in PD . Relevant studies proved that acupuncture can supplement dopamine by maintaining the stability of dopamine in the substantia nigra striatum so as to avoid hyperactivity or lack of dopamine [30].At present, anxiety is often neglected as one of the non-motor symptoms of PD, and there is no drug for the treatment of PDA; hence, anti-anxiety drugs and antidepressant drugs are mainly used. However, antidepressant drugs, such as serotonin reuptake inhibitors (SSRI) combined with anti-Parkinson drugs, such as monoamine oxidase B inhibitors (MAO-BI), can easily aggravate tremor symptoms[31]. There is a lack of clinical evidence regarding the role of anti-anxiety drugs in PDA, only anecdotal evidence exists. Patients with anxiety benefit from more effective and safer forms of complementary and alternative interventions[32.]Acupuncture for patients with PDA has not been intensively investigated. Here, we evaluated the effectiveness of acupuncture for the management of PDA. To our knowledge, this is the first randomized controlled trial of the effectiveness of an acupuncture treatment regimen targeted for PDA. This study preliminarily explored the application of alternative therapy in PDA. Both the RA and the SA groups in the present study showed statistically improvements in anxiety. The results indicated

that there was no significant variance in the degree of improvement between the RA and SA groups after acupuncture. However, we found that two months after the treatment, the statistically improvement in the anxiety and mental status of the participants in the RA group was better than that of those in the SA group. In addition to statistical differences, whether a treatment method has clinical significance is more paramount for patients.The minimal clinically significant difference(MCID) is principal for interpretation of clinical outcomes and clinical decision-making. There is no consensus on MCID of HAM-A[33].

We used anchor based method to calculate MCID of HAM-A20 (supplement 1). Results shows MCID of HAM-A is 4. Therefore, Both the RA （65.6%）and the SA groups（62.5%）in the present study reached MCID and showed clinically improvements in anxiety at the end of treatment.At the end of follow-up , 86.8% patients in RA group and 6.4% patients in SA group reached MCID.

The clinically improvement in the anxiety of the participants in the RA group was better than SA group .It can be preliminarily concluded that although there is a certain placebo effect in the short term, acupuncture is clinically effective on anxiety in patients with PD. The placebo effect of acupuncture in the present study disappeared over time, its therapeutic effect was maintained long-term.

There are two main reasons for this. First, all the participants of this study are Chinese. Acupuncture, as a traditional Chinese therapy, is highly recognized in China. Thus, participants would generally believe that they have received “an effective treatment.” Secondly, as anxiety is a subjective symptom, it is easy to produce a placebo effect during its treatment by using a highly recognized treatment. Thus, to confirm the therapeutic effect of acupuncture in terms of its mechanism, we used ELISA to measure the serum levels of CORT and ACTH in the two groups. CORT and ACTH influence the HPA axis and indirectly reflect a state of anxiety.

Cort and ACTH may play an important role in PDA by interacting with Serotonin(5-HT)receptors[34] .Deficiency of dopamine in PD and excessive dopamine intake results in suppression the synthesis and release of the 5-HT neurons and thereby aggravates the PDA[35]. Imbalance of 5-HT is a pivotal factor in the pathogenesis of PDA. 5-HT reuptake inhibitors are thought to be potentially effective for PDA[36].

We proved that there was no significant variance between the serum levels of CORT in the two groups, whereas the alterations in ACTH levels proved significant between the groups. The serum ACTH levels in the RA group were lower than in the SA one. It may be preliminarily confirmed that acupuncture can reduce the level of ACTH in serum, a finding that is in line with previous results[37], where the authors have proved that acupuncture can alleviate increased stress hormone levels and mitigate anxiety.

Over activation of HPA axis will result in a depletion of 5-HT receptors[38]. Therefore, we can infer that acupuncture can reduce 5-HT depletion by inhibiting the hyperactive HPA axis.This hypothesis is consistent with previous studies .Jing-jing Le verified acupincture could enhance hippocampal 5-HT/5-HT1A by modulating HPA axis[39] .This mechanism may explain the “prolonged effect” of acupuncture two months after the end of treatment. Patients in SA group may temporarily inhibit the over activation of HPA axis through the idea of "I have received effective treatment", but this effect gradually disappears after the treatment. SA cannot have a positive effect on 5-HT.

However, it should be noted that ACTH is an upstream substance of CORT[40]. The decrease in ACTH level did not cause a decrease in the serum level of CORT, which does not mean that acupuncture can reduce anxiety by inhibiting over-activation of the HPA axis. There are some plausible reasons for this. First, the individual differences between the patients were large, the sample size was small, and the preservation and utilization of the ELISA kits may induce bias in the results. Second, the follow-up

duration of this study is short, which may allow for observation of preliminary changes in ACTH level, but not significant changes in CORT level.

As with the primary outcome, participants in both the RA and SA groups showed improvements in their overall conditions and QOL. However, there was no substantial variance between the improvements recorded in the two groups. This may be because variations in UPDRS I score lead to fluctuations in the total UPDRS score. In addition, reduction of anxiety symptoms leads to reduction of motor symptoms. Several movement disorders, such as gait instability and frozen gait, which often occur after anxiety onset, have been proven to be related to the severity of anxiety.5 Improvement of anxiety may also improve the QOL as a whole by promoting the recovery of social roles, and reducing the limitations caused by PD. Two months after the end of treatment, the improvement of the overall condition and QOL of the participants in the RA group was better than that of those in the SA group. Thus, it can be preliminarily concluded that acupuncture can improve the overall condition and QOL of patients with PD by alleviating anxiety symptoms. Anxiety, as a tricky non-motor symptom, is a huge encounter in PD treatment. CBT is a non-drug therapy commonly used to treat PDA[41].There is evidence that acupuncture is comparable to CBT[42]. However, it is well known that patients are likely voluntarily accept routine medical treatment in environments they seem to feel less pressure.28,15 In addition, this study verified acupuncture is an operative substitute technique for the therapy of PDA. The mechanism behind its effect may be related to reduction in the serum level of ACTH. Acupuncture may have a placebo effect in the treatment of anxiety in PD; however, this placebo effect may weaken over time.

This study has some strengths. First, this was a double-blind trial conducted through blinding of patients and acupuncture operator. Second, we administered acupuncture at a 15° angle and a 90° angle. The current sham needle could not be used to achieve this multi-angle needle entry. The sham needle we developed and used in this study was highly consistent with the real acupuncture needle in terms of shape, angle, and feel. Its masking effect was good, which can ensure the validity of the data. Third, this research provides a basis for the development of effective alternative therapies for PDA. Although the placebo effect of acupuncture exists, its safety and tolerance are widely accepted by patients.

Limitations

This study has some limitations as well. Regarding evaluation, some items of the HAM-A scale are experienced by Parkinson's disease patients with anxiety and those without anxiety. Thus, there may be some bias in using HAM-A score ≥ 14 as the standard for evaluating anxiety in PD. Therefore, more precise criteria are needed to evaluate anxiety in PD. Regarding participants, only Chinese participants were included in this study. Thus, the placebo effect may have been caused by cultural factors. In future study, cultural differences should be taken into account, and multi center research should be used to further confirm the efficacy of acupuncture in the treatment of PDA. Economic benefits, patient acceptance, and feasibility should be also considered in future study to evaluate application value of acupuncture .

Conclusions

This randomized double-blind controlled trial confirmed that acupuncture for 8 weeks can effectively ameliorated the anxiety of PD patients , and its mechanism may be related to the inhibition of HPA axis hyperactivity. Additionally , acupuncture can improve overall motor functions and well-being of PD patients by ameliorating the anxiety.Future research is needed to determine more appropriate methods for the diagnosis of PDA and to confirm the effectiveness and appliance of acupuncture for PDA therapy with broad sample size and objective indicators.

To reduce bias, we used a rigorous allocation method with blinded patients, evaluators, and statistical analysts. We also used a new type of sham acupuncture tool to avoid the placebo effect. Due to the particularity of acupuncture operation, the previous sham needle intervention can only be blinded to patients, but not the operator. In this study, the opaque base and tube were used to blind the operator. Therefore, this study realized the blinding of patients and operators at the same time.

This trial potentially provided valid clinical evidence. The findings of this study validated the effects and mechanism of acupuncture as a complementary treatment method for PDA. The study had the potential to provide strong clinical evidence as well as new guidelines for the treatment of PDA.

10 Ethical Aspects

The present study protocol complies with the requirements of the following directives and guidelines:

- Ethical review measures for biomedical research involving human beings,(2016)
- Quality management specification for clinical trials of medical devices,(2016)
- WMA, Helsinki Declaration
- International ethical guide to biomedical research on human body

Informed consent for clinical study

Project name: observation of clinical efficacy of acupuncture treatment in Parkinson's anxiety

Applicant (application department): Parkinson's clinic

Clinical trial institution: The First Affiliated Hospital of Guangzhou University of Traditional Chinese Medicine

Version number or version date: version 3.0

Principal investigator: Lixing Zhuang

Personal reading materials

Dear patients, please read this article carefully and you are welcome to ask questions and discuss them with your family, relatives, friends or us.

You are now invited to participate in this clinical study.The purpose of the study is to prove the clinical effect of acupuncture on Parkinson's anxiety by comparing acupuncture with fake acupuncture, and to provide technical and evidence-based medicine for the treatment of Parkinson's anxiety.

Whether to participate in this study depends entirely on your wishes, and please read this material in detail before you make a decision.It helps you to have a comprehensive understanding of the purpose, methods, the research process, the possible benefits and inconveniences of participating in the study, and your interests.The information provided to you by this informed consent form can help you decide whether to participate in this clinical trial. Please ask any questions from the investigator responsible for the project trial, or discuss it with your family, relatives and friends to ensure that you fully understand the content.Whether you participate in this trial is voluntary, and if you agree to participate in this clinical trial, please sign the statement in the informed consent form.

1、 What kind of study is this?[Research Background and Purpose]

The study is the clinical effect of acupuncture for Parkinson's Disease Anxiety (PDA).To compare the clinical efficacy of the two therapies for Parkinson's anxiety through acupuncture and fake acupuncture methods, and to provide technical and evidence-based medicine basis for the treatment of Parkinson's anxiety.

In the previous clinical study, the improvement effect of "acupuncture method" on the motor symptoms of Parkinson's Disease (PD) was preliminarily found by scoring on the UPDRS and Hoehn-Yahr grading scale.The effect of "acupuncture" on the improvement of anxiety symptoms in

PDA patients was preliminarily found by the HAMA scale score.

Through multimodal MRI examination, it was initially found that acupuncture could activate signal changes in the hippocampus, striatum, and amygdala brain areas in PDA patients to improve PDA.

2、 Was participation in this study voluntary?

Participation in this study is voluntary.You have the right to decide whether to participate in this study and need no reason not to participate in this study.Failure to participate in this study will not suffer any discrimination or retaliation, it will not affect your relationship with your doctor or your medical interests, and you will continue to receive the treatment given by your doctor.

3. Who should not participate in this project research

You will not be eligible for this study if you have any of the following conditions:

(1) It is clear that there are serious and other systemic diseases, such as immune system diseases, endocrine system diseases (such as pheochromocytoma, hyperthyroidism, etc.), respiratory system diseases, cardiovascular and cerebrovascular diseases, or liver and kidney insufficiency;

(2) Patients with a clear history of mental illness or associated with psychotic symptoms;

(3) Fear of acupuncturists;

(4) A history of abuse of certain substances (such as alcohol, drugs and caffeine) in the past half a year;

(5) Those who have participated in other clinical trials such as drugs and acupuncture in the 1 month before their inclusion in the study.

4、 What are the treatments used in this study?

(1) Needle acupuncture group: to the true acupuncture treatment, acupuncture for 3 times a week, acupuncture for 1 week is a course of treatment, continuous treatment for 8 weeks.

(2) Control group: sham acupuncture treatment, 3 times a week of acupuncture, 1 week of acupuncture is a course of treatment, 8 weeks of continuous treatment.

From the start of treatment to the end of treatment, the original effective treatment dose of the patient should be maintained. If the drug dose must be increased or decreased, the case observation form should be truthfully recorded.

5、 What will you need to do if you participate in the study?

If you participate in this study, you enter the screening period after signing the informed consent form. If your doctor thinks you meet the inclusion criteria and are eligible for this study, you will receive treated based on the random number generated by the computer.You will have a 50% chance of entering the treatment group and a 50% chance of entering the control group.The treatment group gave real acupuncture and basic medication. On the basis of basic medication, acupuncture was given 3 times a week for 8 consecutive weeks, with a total of 24 times.The control group received medoba (Dopa hydrazine tablets, Shanghai Roche Pharmaceutical Co., approval No.: Chinese Pharmaceutical quasi-word 1010930498) to maintain the original effective treatment dose of the patient from the start of treatment to the end of treatment.Both treatment and control groups should take 4ml of blood before and after treatment for serum ACTH and the test before and after CORT.

In this study, the cases in the department of the First Affiliated Hospital of Guangzhou University of Traditional Chinese Medicine were collected in the outpatient and rehabilitation centers in strict accordance with the ethical review until June 2022. The total number of cases was 70, with 35 cases in each group.

6、 Possible benefits from participating in the study

You and society will benefit or may not benefit from this study.Such benefits include the potential

for improvement in your condition and this study may help further clarify the use of acupuncture in Parkinson's anxiety for the treatment of patients with a similar condition.

7、 Possible adverse reactions, risks, discomfort, and inconvenience of participating in the study

Possible adverse events of participating in the study mainly include: halo needle, block needle, bending needle, broken needle, bleeding and subcutaneous hematoma, peripheral nerve injury, etc.In case of adverse reactions, the researcher will stop the current treatment in time and take necessary treatment measures. Meanwhile, the medical expenses caused by the adverse reactions will be borne by the research group.

In addition, any treatment may be ineffective, and the disease continues to develop due to ineffective treatment or combined with other diseases.This is the treatment risk that every patient will face, and even if they does not participate in this clinical study.During the study period, if the doctor or investigator finds the treatment measures taken in the study ineffective, the study will be terminated and switched to other treatments that may be effective.

8、 Related fees

The research team will pay your costs related to the study, including: treatment costs, treatment equipment (needles, medical cotton swabs, Ann iodine II skin disinfectant), blood test, biochemical indicators for adrenal corticotrohormone (ACTH), cortisol (CORT), index analysis costs by the research team, and provide a nutritious breakfast on the day of the blood draw. Anti-Parkinson's disease drugs is not free for regular treatment.

If you also have other diseases and are not related to the study, the required treatment and examination, and the cost of switching to other treatment measures because of the study, will not be free of charge.Any research-related damage will be dealt with in accordance with relevant national laws and regulations.

9、 How do you protect your privacy rights?

Your medical records (including research medical records, etc.) will be kept in the hospital as required.Your participation in the study and your personal information in the study are confidential, and the results of the study will not reveal your personal identity.Higher health / drug / research administration, hospital ethics committee, investigators will be allowed to access your medical records to verify procedures and / or data for the clinical study.We will strictly protect the privacy of your personal medical information within the existing laws.

10. Important tips

In order to ensure the reliability of clinical research, I hope you can do the following throughout the clinical research: ① does not receive other similar treatment; for any discomfort during ② research, please report to your doctor in charge.③ like Parkinson's anxiety symptoms when the aggravation should be timely to the specialist visit.

The treatment plan for this study is not the only treatment option for your current disease, so you can consult with your doctor and then decide whether to participate in this study.

11. Can you quit after attending the study?

Whether or not one attends all depends on your willingness.You may refuse to participate in this study or withdraw at any time during the course of the study, neither affecting your relationship with your doctor, nor the loss of your medical or other interests, and you are free from any discrimination or retaliation.

Your doctor may suspend your participation in this study at any time in your best interests.

If you do not participate in this study, or drop out, there are many other alternative treatments.You do not have to participate in this study in order to treat your illness.If you withdraw from the study for any reason, in your best interest, you may be asked about your medication use and you may also be asked to have a laboratory and physical examination if your doctor believes it.

If you choose to participate in this study after full consideration, we want you to continue the whole study process.

12. More information acquisition

You can ask any questions about this study at any time, and your doctor will leave you with contact information to allow you to answer your questions.

Your doctor will inform you promptly if there is any important new information during the study that may affect your willingness to continue attending the study.

13. What are you supposed to do right now?

It is up to you to participate in this study.You can also discuss it with your family before making a decision.Before you make your decision to participate in the study, please ask your doctor any questions until you fully understand the study.

Finally, thank you for reading the above materials.If you decide to participate in this study, please tell your doctor that they will arrange for you about the study.Please keep this information.If you have any questions about your rights in this study, please contact the Ethics Committee of the Center at 020-36588667 or 020-36591965; Email: gztcmlunli@163.com; Fax: 020-36591346.

REFERENCES

1. Tolosa E, Garrido A, Scholz SW, Poewe W. Challenges in the diagnosis of Parkinson's disease. *Lancet Neurology.* 2021;20(5):385-397. doi:10.1016/S1474-4422(21)00030-2
2. Dissanayaka NNNW, White E, O'Sullivan JD, Marsh R, Pachana NA, Byrne GJ. The Clinical Spectrum of Anxiety in Parkinson's Disease. *Movement Disorders.* 2014;29(8):967-975. doi:10.1002/mds.25937
3. Broen MPG, Narayen NE, Kuijf ML, Dissanayaka NNW, Leentjens AFG. Prevalence of anxiety in Parkinson's disease: A systematic review and meta-analysis. *Movement Disorders.* 2016;31(8):1125-1133. doi:10.1002/mds.26643
4. Zhu K, van Hilten JJ, Marinus J. Onset and evolution of anxiety in Parkinson's disease. *European Journal of Neurology.* 2017;24(2):404-411. doi:10.1111/ene.13217
5. Bandelow B, Lichte T, Rudolf S, Wiltink J, Beutel ME. The German guidelines for the treatment of anxiety disorders. *European Archives of Psychiatry and Clinical Neuroscience.* 2015;265(5):363-373. doi:10.1007/s00406-014-0563-z
6. Dissanayaka NNW, White E, O'Sullivan JD, et al. Characteristics and Treatment of Anxiety Disorders in Parkinson's Disease. *Movement Disorders Clinical Practice.* 2015;2(2):155-162. doi:10.1002/mdc3.12157
7. Martens KAE, Hall JM, Gilat M, Georgiades MJ, Walton CC, Lewis SJG. Anxiety is associated with freezing of gait and attentional set-shifting in Parkinson's disease: A new perspective for early intervention. *Gait Posture.* 2016;49:431-436. doi:10.1016/j.gaitpost.2016.07.182
8. Yang S, Sajatovic M, Walter BL. Psychosocial Interventions for Depression and Anxiety in

Parkinson's Disease. *Journal of Geriatric Psychiatry and Neurology.* 2012;25(2):113-121. doi:10.1177/0891988712445096

9. Chen FP, Chang CM, Shiu JH, et al. A Clinical Study of Integrating Acupuncture and Western Medicine in Treating Patients with Parkinson's Disease. *American Journal of Chinese Medicine.* 2015;43(3):407-423. doi:10.1142/S0192415x15500263
10. Iseki C, Furuta T, Suzuki M, et al. Acupuncture Alleviated the Nonmotor Symptoms of Parkinson's Disease including Pain, Depression, and Autonomic Symptoms. *Case Rep Neurol Med.* 2014;2014:953109. doi:10.1155/2014/953109
11. Antonini A, Moro E, Godeiro C, Reichmann H. Medical and Surgical Management of Advanced Parkinson's Disease. *Movement Disorders.* 2018;33(6):900-908. doi:10.1002/mds.27340
12. Chen HB, Chen SD, Li SH. Diagnostic criteria and treatment guidelines for depression, anxiety and psychotic disorders in Parkinson’s disease. *Chinese Journal of Neurology.* 2013;46(1):56-60. doi:10.3760/cma.j.issn.1006-7876.2013.01.015
13. Cook SC, Schwartz AC, Kaslow NJ. Evidence-Based Psychotherapy: Advantages and Challenges. *Neurotherapeutics.* 2017;14(3):537-545. doi:10.1007/s13311-017-0549-4
14. Li M, Xing X, Yao L, et al. Acupuncture for treatment of anxiety, an overview of systematic reviews. *Complementary Therapies in Medicine.* 2019;43:247-252. doi:10.1016/j.ctim.2019.02.013
15. Craske MG, Stein MB. Anxiety. *Lancet.* 2016;388(10063):3048-3059. doi:10.1016/S0140-6736(16)30381-6
16. Errington-Evans N. Acupuncture for anxiety. *CNS Neurosci Ther.* 2012;18(4):277-284. doi:10.1111/j.1755-5949.2011.00254.x
17. Yang XY, Yang NB, Huang FF, Ren S, Li ZJ. Effectiveness of acupuncture on anxiety disorder: a systematic review and meta-analysis of randomised controlled trials. *Annals of General Psychiatry.* 2021;20(1). doi:10.1186/s12991-021-00327-5
18. Wen X, Li K, Wen H, et al. Acupuncture-Related Therapies for Parkinson's Disease: A Meta-Analysis and Qualitative Review. *Frontiers in Aging Neuroscience.* 2021;13:676827. doi:10.3389/fnagi.2021.676827
19. Li Z, Chen J, Cheng J, et al. Acupuncture Modulates the Cerebello-Thalamo-Cortical Circuit and Cognitive Brain Regions in Patients of Parkinson's Disease With Tremor. *Frontiers in Aging Neuroscience.* 2018;10:206. doi:10.3389/fnagi.2018.00206
20. Fukuda S, Egawa M. Effect of Acupuncture on Gait in Parkinson—s Disease: A Case Report. *Acupuncture in Medicine.* 2015;33(4):325-328. doi:10.1136/acupmed-2015-010760
21. Toosizadeh N, Lei H, Schwenk M, et al. Does integrative medicine enhance balance in aging adults? Proof of concept for the benefit of electroacupuncture therapy in Parkinson's disease. *Gerontology.* 2015;61(1):3-14. doi:10.1159/000363442
22. Zeng BY, Zhao K. Effect of acupuncture on the motor and nonmotor symptoms in Parkinson's disease—a review of clinical studies. *CNS Neuroscience & Therapeutics.* 2016;22(5):333-341. doi:10.1111/cns.12507
23. McGeeney BE. Acupuncture is all placebo and here is why. *Headache.* 2015;55(3):465-469. doi:10.1111/head.12524
24. Ju L, Wu X, Xu D, Fei L, Ning H, Sun J. Application of placebo acupuncture in randomized controlled trials in the past 10 years in foreign countries. *Chin Acupunct Moxibustion.*

2016;36(2):203-206. doi:10.13703/j.0255-2930.2016.02.029

25. Liu B. Establishing clinical efficacy evaluation system to promote acupuncture internationalization. *Chin Acupunct Moxibustion.* 2018;38(5):545-546. doi:10.13703/j.0255-2930.2018.05.025

26. Lee MS, Shin BC, Kong JC, Ernst E. Effectiveness of acupuncture for Parkinson's disease: a systematic review. *Mov Disord.* 2008;23(11):1505-1515. doi:10.1002/mds.21993

27. Bloem BR, Okun MS, Klein C. Parkinson's disease. *Lancet.* 2021;397(10291):2284-2303. doi:10.1016/S0140-6736(21)00218-X

28. Fan JQ, Xu ZQ, Chen YY, et al. Efficacy of Acupuncture for Parkinson's Disease Anxiety: Two-Stage Protocol for a Randomized Controlled Clinical Trial. *Evid Based Complement Alternat Med.* 2022;2022:5180193. doi:10.1155/2022/5180193

29. Khatri DK, Choudhary M, Sood A, Singh SB. Anxiety: An ignored aspect of Parkinson's disease lacking attention. *Biomed Pharmacother.* 2020;131:110776. doi:10.1016/j.biopha.2020.110776

30. Kim S-T, Moon W, Chae Y, Kim YJ, Lee H, Park H-J. The effect of electroaucpuncture for 1-methyl-4-phenyl-1, 2, 3, 6-tetrahydropyridine-induced proteomic changes in the mouse striatum. *J Physiol Sci.* 2010;60(1):27-34. doi:10.1007/s12576-009-0061-7

31. Chen H, Chen S, Li S. Diagnostic criteria and treatment guidelines for depression, anxiety and psychotic disorders in Parkinson' s disease. *Chin J Neurol.* 2013;46(1):56-60. doi:10.3760/cma.j.issn.1006-7876.2013.01.015

32. Li M, Xing X, Yao L, et al. Acupuncture for treatment of anxiety, an overview of systematic reviews. *Complement Ther Med.* 2019;43:247-252. doi:10.1016/j.ctim.2019.02.013

33. McGlothlin AE, Lewis RJ. Minimal clinically important difference: defining what really matters to patients. *JAMA.* 2014;312(13):1342-1343.

34. Seo SY, Bang SK, Kang SY, Cho SJ, Choi KH, Ryu YH. Acupuncture Alleviates Anxiety and 22-kHz Ultrasonic Vocalizations in Rats Subjected to Repeated Alcohol Administration by Modulating the Brain-Derived Neurotrophic Factor/Corticotropin-Releasing Hormone Signaling Pathway. Int J Mol Sci. 2021;22(8):4037. doi:10.3390/ijms22084037

35. Pilar-Cuéllar F, Vidal R, Díaz Á, et al. Enhanced Stress Response in 5-HT(1A)R Overexpressing Mice: Altered HPA Function and Hippocampal Long-Term Potentiation. ACS Chem Neurosci. 2017;8(11):2393-2401. doi:10.1021/acschemneuro.7b00156

36. Khatri DK, Choudhary M, Sood A, Singh SB. Anxiety: An ignored aspect of Parkinson's disease lacking attention. Biomed Pharmacother. 2020;131:110776.

37. Seo SY, Bang SK, Kang SY, Cho SJ, Choi KH, Ryu YH. Acupuncture Alleviates Anxiety and 22-kHz Ultrasonic Vocalizations in Rats Subjected to Repeated Alcohol Administration by Modulating the Brain-Derived Neurotrophic Factor/Corticotropin-Releasing Hormone Signaling Pathway. Int J Mol Sci. 2021;22(8):4037. doi:10.3390/ijms22084037.

38. Pilar-Cuéllar F, Vidal R, Díaz Á, et al. Enhanced Stress Response in 5-HT(1A)R Overexpressing Mice: Altered HPA Function and Hippocampal Long-Term Potentiation. ACS Chem Neurosci. 2017;8(11):2393-2401. doi:10.1021/acschemneuro.7b00156.

39. Le J-j, Yi T, Qi L, Li J, Shao L, Dong J-C. Electroacupuncture regulate hypothalamic – pituitary – adrenal axis and enhance hippocampal serotonin system in a rat model of depression. Neurosci Lett. 2016;615:66-71.

40. Frankiensztajn LM, Elliott E, Koren O. The microbiota and the

hypothalamus-pituitary-adrenocortical (HPA) axis, implications for anxiety and stress disorders. Curr Opin Neurobiol. 2020;62:76-82. doi:10.1016/j.conb.2019.12.003

41. Moonen AJH, Mulders AEP, Defebvre L, et al. Cognitive Behavioral Therapy for Anxiety in Parkinson's Disease: A Randomized Controlled Trial. Mov Disord. 2021;36(11):2539-2548. doi:10.1002/mds.28533.

42. Chae Y, Yeom M, Han JH, et al. Effect of acupuncture on anxiety-like behavior during nicotine withdrawal and relevant mechanisms. Neurosci Lett. 2008;430(2):98-102. doi:10.1016/j.neulet.2007.10.026.
