# Supplementary material for: Effectiveness of Acupuncture for Anxiety Among Patients With Parkinson Disease: A Randomized Clinical Trial
Source: JAMA Netw Open. 2022 Sep 21;5(9):e2232133. doi: 10.1001/jamanetworkopen.2022.32133 (PMC9494193; doi:10.1001/jamanetworkopen.2022.32133)
Supplement: Supplement 2. — eFigure 1. The Relationship Between PD and PDA eFigure 2. Selection and Location of the Acupoints for Acupuncture eAppendix. The Masking Effect of Sham Acupuncture eFigure 3. Placebo Acupuncture Appliance Diagram eFigure 4. Different Bases of the Appliance eFigure 5. Placebo Acupuncture on the Head eTable 1. Primary and Secondary Outcomes eTable 2. Levodopa Equivalent Dose Conversion eTable 3. Operation Rules of Real Acupuncture eTable 4. VAS Scores [M (P25, P75)] eTable 5. Cohen’s Kappa of Sham Acupuncture eReferences [file jamanetwopen-e2232133-s002.pdf]

Supplemental Online Content

Fan JQ, Lu WJ, Tan WQ, et al. Effectiveness of acupuncture for anxiety among patients with Parkinson disease: a randomized clinical trial. *JAMA Netw Open.* 2022;5(9):e2232133. doi:10.1001/jamanetworkopen.2022.32133

eFigure 1. The Relationship Between PD and PDA

eFigure 2. Selection and Location of the Acupoints for Acupuncture

eAppendix. The Masking Effect of Sham Acupuncture

eFigure 3. Placebo Acupuncture Appliance Diagram

eFigure 4. Different Bases of the Appliance

eFigure 5. Placebo Acupuncture on the Head

eTable 1. Primary and Secondary Outcomes

eTable 2. Levodopa Equivalent Dose Conversion

eTable 3. Operation Rules of Real Acupuncture

eTable 4. VAS Scores [M (P25, P75)]

eTable 5. Cohen's Kappa of Sham Acupuncture

eReferences

This supplemental material has been provided by the authors to give readers additional information about their work.

© 2022 Fan JQ et al. *JAMA Network Open.*

eTable 1 Primary and secondary outcomes

Domain	Instrument
Anxiety state	Hamilton Anxiety Scale[a]
Mental Status	Unified Parkinson's Disease Rating Ⅰ[b]
Emotional well-being	39-item Parkinson Disease Questionnaire-Emotional well-being[c]
Clinical assessment	Unified Parkinson's Disease Rating[d]
Activities of daily living	39-item Parkinson Disease Questionnaire-Activities of daily living[e]
Quality of life	39-item Parkinson Disease Questionnaire[f]
Blood serum levels of ACTH	ELISA Kit of adrenocorticotropic hormone[g]
Blood serum levels of CORT	ELISA Kit of cortisol[h]

[a] Scores range from 0 to 56, with higher scores indicating more serious anxiety.
[b] Scores range from 0 to 16, with higher scores indicating worse mental status.
[c] Scores range from 0 to 28, with higher scores standing for worse emotional well-being.
[d] Scores range from 0 to192, with higher scores indicating more severe overall symptoms of Parkinson's disease.
[e] Scores range from 0 to 20, with higher scores indicating worse activities of daily living.
[f] Scores range from 0 to156, with higher scores indicating worse quality of life.
[G] Higher content indicates more activitation of the HPA axis.
[h] Higher content indicates more activitation of the HPA axis.

© 2022 Fan JQ et al. *JAMA Network Open.*

eTable 2 Levodopa equivalent dose conversion

There are many kinds of anti Parkinson drugs. Patients often take multiple anti Parkinson drugs at the same time, so it is impossible to evaluate the baseline situation of patients before treatment. In this study, the calculation software of levodopa equivalent dose was used to convert different kinds of anti Parkinson drugs taken by patients into levodopa equivalent dose.

Drug	Conversion
L-dopa	1
L-dopa CR	0.75
L-dopa + Entacapone	1.33
L-dopa + Opicapone	1.7
L-dopa + Tolcapone	1.5
Rotigotine	30
Ropinirole	20
Pramipexole	100
Selegiline	10
Rasagiline	100
Apomorphine	10

eTable 3 Operation rules of Real acupuncture

	Needle specification	Acupuncture angle	Acupuncture depth
Si Shen Zhen	0.25 × 25 mm	45 degree angle	15-20mm
GV 24 (shen ting)	0.25 × 25 mm	45 degree angle	15-20mm
GV 29 (yin tang)	0.25 × 25 mm	45 degree angle	15-20mm
HT7(shen men)	0.25 × 25 mm	90 degree angle	15-20mm
SP 6 (*san yin -jiao*)	0.25 × 40 mm	90 degree angle	25-30mm

© 2022 Fan JQ et al. *JAMA Network Open.*

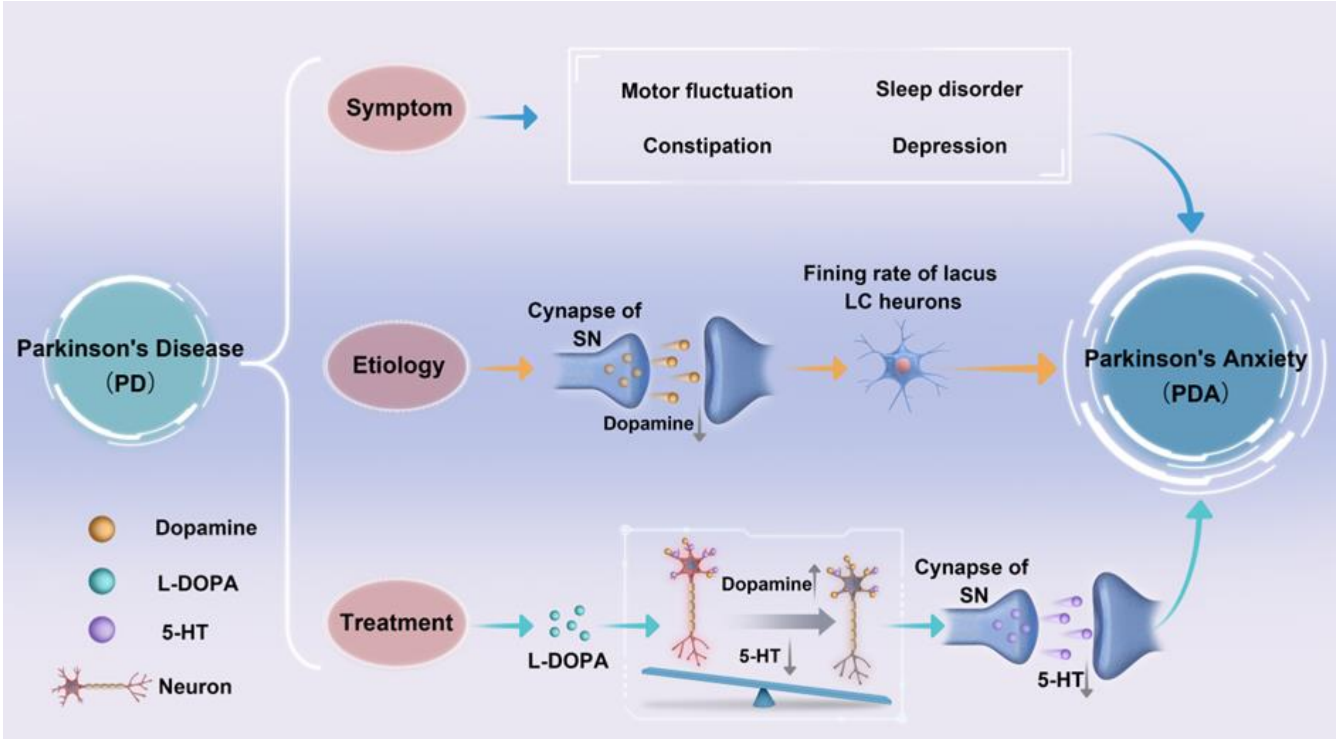

eFigure 1. The relationship between PD and PDA.

© 2022 Fan JQ et al. *JAMA Network Open.*

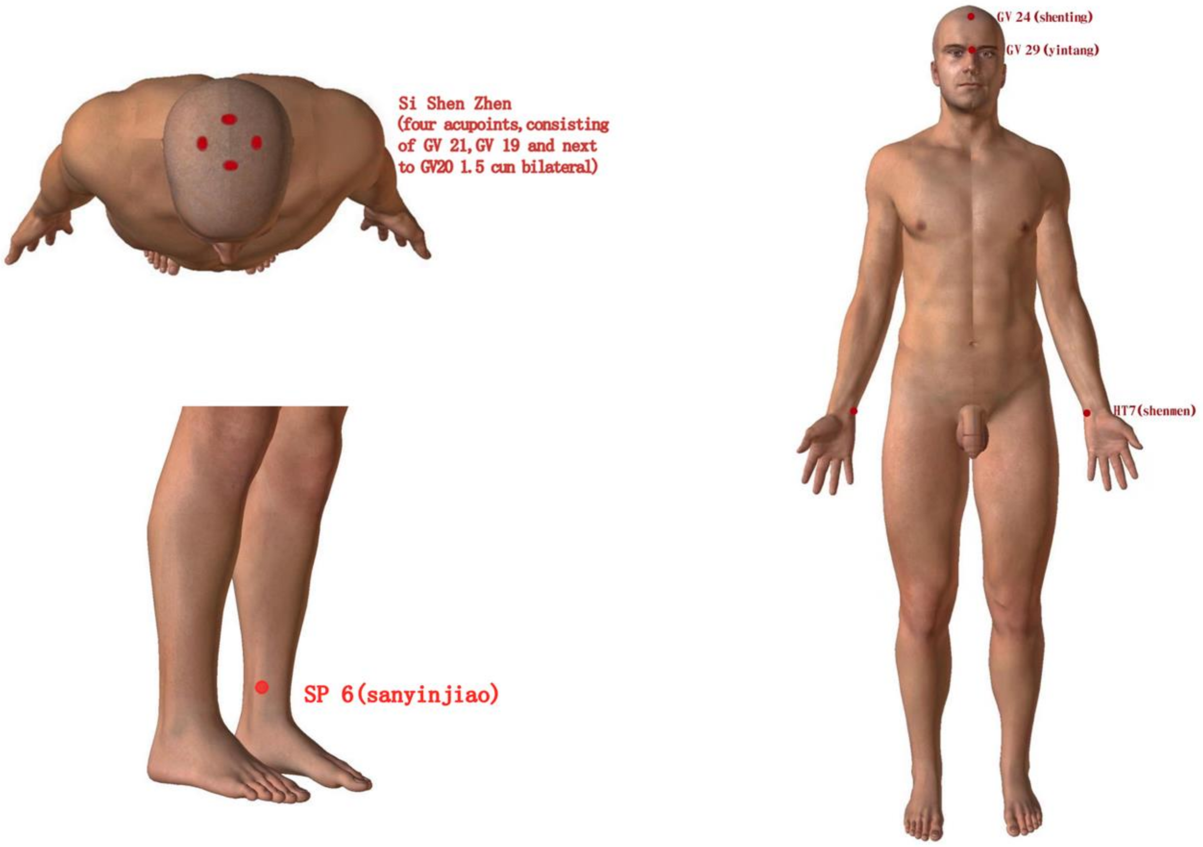

eFigure 2 Selection and location of the acupoints for acupuncture

© 2022 Fan JQ et al. *JAMA Network Open.*

eAppendix 1

The masking effect of sham acupuncture

1. Introduction

Acupuncture, one of the essential therapeutic methods of traditional Chinese Medicine, is used in many clinical practices in China and overseas. As the interest of acupuncture develop in the world, a great deal of acupuncture clinical studies were conducted. However, some systematic review showed that many acupuncture clinical trials are of low evidence-based for they are out of blinding[1-4]. Non-blinding gives rise to expected bias, and placebo effects[5] are inevitably existed which make trials less convincing. Thus, a practical placebo acupuncture appliance is urgent needed.

The first reported placebo acupuncture appliance[6], designed by Streitberger and Kleinhenz,was covered by a plastic sheet so that patients cannot see whether the needle was inserted or not . Nobuari Takakura and Hiroyoshi Yajima modified the placebo appliance with an opaque tube[7] . The two placebo appliances are still popular and used in many randomized trials[8-10] but they can just insert straight without changing the angle. Therefore, these appliances cannot fix different acupoints[11] such as Baihui (DU20), which stands on the head and needs inclined angle. Given the present defective placebo acupuncture appliances, designing a new practical placebo acupuncture appliance that matches different inserted angle is pressing.

© 2022 Fan JQ et al. *JAMA Network Open.*

2. A new practical placebo acupuncture appliance

To match acupuncture clinical study practically, our team designed a placebo acupuncture appliance, which can adjust different needling angles. The appliance has already granted the patent by China national intellectual property administration (No. ZL202121352221.7). The placebo acupuncture appliance contains a pedestal and a tube. The pedestal, made by resin material, is opaque so that it can isolate patients' eyesight,and they cannot visually decide whether the needle is inserted into skin or not. There is a sticky patch in the base of the pedestal to ensure the pedestal can stick to the skin. The tube is transparent so that it is convenient for doctors to insert the needle through the tube. In addition, we designed two types of pedestals. One is hollowed in the base, the other is not hollowed . For the sake of real

© 2022 Fan JQ et al. *JAMA Network Open.*

acupuncture, we use the hollowed appliance, and the needle can insert into the skin directly and hygienically. For the sake of placebo acupuncture, we use the non-hollowed appliance and the blunt needle. The sticky base can clamp the non-invasive needle so that the needle will not drop whenever patients move. For different angles, there are three holes in the pedestal, one on the top and two on the sides. If putting the tube in the top hole, then the needle can hold a straight angle while putting the tube in the side hole, the needle is beveled to the skin that can match the need of needling in scalp (Figure 5).

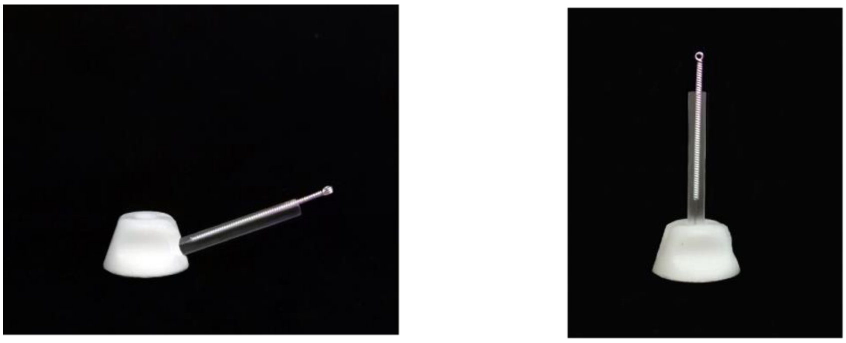

Needle at a 15 degree angle **Needle at a 90 degree angle**

eFigure. 3. placebo acupuncture appliance diagram

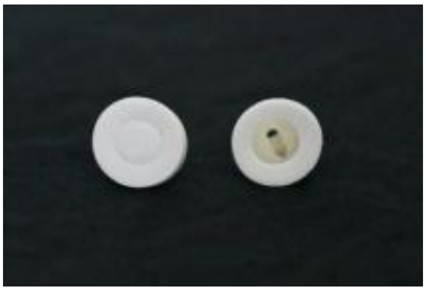

eFigure. 4. different bases of the appliance

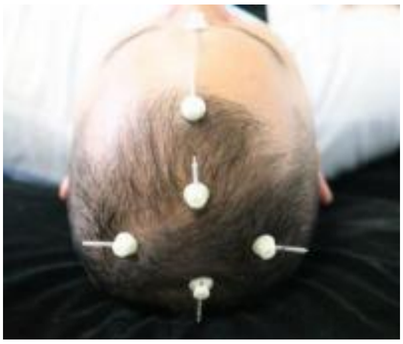

eFigure. 5. placebo acupuncture on the head

© 2022 Fan JQ et al. *JAMA Network Open*

3. Protocol for masking effect of sham acupuncture

In order to testify the masking effect, we recruited 60 volunteers, aged 24 to 66, with no sign of disease,to test whether they can judge the type of acupuncture they received. All these volunteers have experience of acupuncture treatment and was told that they would test a new acupuncture appliance. This pilot study is approved by the Ethics Committee of the First Affiliated Hospital of Guangzhou University of Chinese Medicine (No.K[2021]015).

The study was conducted by two experienced acupuncture therapists,graduated from Guangzhou University of Chinese Medicine and have clinical experience in the First Affiliated Hospital of Guangzhou University of Chinese Medicine over two years. Each volunteer with a patch over their eyes and was randomized received real or placebo acupuncture in scalp and limbs, radial and ulnar, such as Shenting (DU24), Hegu (LI4) and Chize (LU5) to simulate clinical scene in reality. After disinfection the acupoints, we sticked the pedestals to volunteers' skin and chose sharp or blunt needles via guiding tube according to the allocation. We removed the needles 2 minutes later. Once the needles removed, we asked volunteers how they felt by visual analogue scale (VAS), testing the feeling of acupuncture, and which type of acupuncture did they believed they received .

© 2022 Fan JQ et al. *JAMA Network Open*

4.Results

The outcome showed that there is no VAS score significant difference between real acupuncture group and placebo acupuncture group. The Cohen' s Kappa coefficient of two groups was -0.033(95% CI:0 to 0.28). No statistically significant difference either.

eTable 4 VAS scores [M (P25, P75)]

	Real acupuncture group（n=30）	Placebo acupuncture group（n=30）	*Z*	*P*
VAS score	4.50(2.00,7.75)	6.00(2.00,8.00)	-0.724	0.469

VAS scores of two groups did not conform to normal distribution (Shapiro-Wilk=0.903, 0.889, *P*=0.010, 0.005), so mann-Whitney U test was used for analysis, and data results were reported by median and quartile.

eTable 5 Cohen's kappa of sham acupuncture

	Believed received real acupuncture	Believed received placebo acupuncture	*κ*	*P*	95%CI
Real acupuncture group（n=30）	13(43.33%)	17(56.67%)	0.033	0.793	0,0.28
Placebo acupuncture group（n=30）	12(40.00%)	18(60.00%)			

© 2022 Fan JQ et al. *JAMA Network Open*

5.Discussion

Based on the result, we consider the new placebo acupuncture appliance has a good masking effect. On the one hand, volunteers felt the same no matter what type of acupuncture they received. But the pain volunteers felt fluctuates, we thought it is due to the different pain tolerant level of different people. On the other hand, consistency was evaluated as perfect if κ was > 0.8, good if 0.6 < $\kappa \leqslant$ 0.8, moderate if 0.4 < $\kappa \leqslant$ 0.6, fair if 0.2 < $\kappa \leqslant$ 0.4, and poor if κ was $\leqslant$ 0.2[12]. The Cohen's kappa less than 0.2 so that the consistency between the type of acupuncture volunteers received and the type they thought was quite poor. Thus,we can draw a conclusion that volunteers cannot distinguish what type of acupuncture they received. The new placebo appliance has a good masking effect.

CONSORT[13] statement and its extensive acupuncture statement, STRICTA, are used for evaluating the quality of randomized controlled trials (RCTs). Since the two statements has introduced in China, the quality of acupuncture RCTs improved[14]. Trials protocols can be modified according to the two statements, but the perceived difficulty of acupuncture RCTs is blinding. CONSORT and STRICTA statements mentioned blinding as one of the important items when evaluating RCTs. If blinding in acupuncture can be realized,the quality and evidence-based level of acupuncture RCTs will improve. The new placebo acupuncture appliance can switch different inserted angles to meet the need of acupoints in scalp. The pilot study showed that the appliance blinds patients practically and is simple to use. Any trained researchers can use it with

© 2022 Fan JQ et al. *JAMA Network Open*

no difficulty. Given the good masking effect and simple usage, the new practical placebo acupuncture appliance can meet the requirements of acupuncture RCTs and improve the level of evidence. Considering that patients' expectation has a great impact on treatment effect[15] and doctors' expertise rather than reported bias influence acupuncture's effectiveness[11] , we focus on reduce the bias of expectation. The new placebo acupuncture appliance is practical in clinical trials[16], we will put our best foot forward to promoting it and help to improve the evidence-based level.

eReference

1. Hammerschlag R, Milley R, Colbert A, Weih J, Yohalem-Ilsley B, Mist S, et al. Randomized Controlled Trials of Acupuncture (1997-2007): An Assessment of Reporting Quality with a CONSORT- and STRICTA-Based Instrument. Evidence-based complementary and alternative medicine :eCAM 2011; 2011.
2. Liu X, Xu Z, Wang Y, Luo H, Zou D, Zhou Z, et al. Evaluating the Quality of Reports About Randomized Controlled Trials of Acupuncture for Low Back Pain. Journal of pain research 2021; 14: 1141-51.
3. Lu T, Lu C, Li H, Xing X, Deng X, Li X, et al. The reporting quality and risk of bias of randomized controlled trials of acupuncture for migraine: Methodological study based on STRICTA and RoB 2.0. Complementary therapies in medicine 2020; 52: 102433.
4. Zhuang L,He J,Zhuang X,Lu L. Quality of reporting on randomized controlled trials of acupuncture for stroke rehabilitation. BMC complementary and alternative medicine 2014; 14: 151.
5. Beecher HK. The powerful placebo. J Am Med Assoc 1955; 159(17): 1602-6.
6. Streitberger K,Kleinhenz J. Introducing a placebo needle into acupuncture research. Lancet 1998; 352(9125): 364-5.
7. Takakura N, Yajima H. A double-blind placebo needle for acupuncture research. BMC complementary and alternative medicine 2007; 7: 31.
8. Barth J, Muff S, Kern A, Zieger A, Keiser S, Zoller M, et al. Effect of Briefing on Acupuncture Treatment Outcome Expectations, Pain, and Adverse Side Effects Among Patients With Chronic Low Back Pain: A Randomized Clinical Trial. JAMA network open 2021; 4(9): e2121418.
9. Birch S, Lee M, Kim T, Alraek T. Historical perspectives on using sham acupuncture in acupuncture clinical trials. Integrative medicine research 2022; 11(1): 100725.
10. Zyczynski H, Richter H, Sung V, Lukacz E, Arya L, Rahn D, et al. Percutaneous Tibial Nerve Stimulation vs Sham Stimulation for Fecal Incontinence in Women: NeurOmodulaTion for Accidental Bowel Leakage Randomized Clinical Trial. The American journal of gastroenterology 2022; 117(4): 654-67.
11. Fei Y, Cao H, Xia R, Chai Q, Liang C, Feng Y, et al. Methodological challenges in design and conduct of randomised controlled trials in acupuncture. BMJ (Clinical research ed) 2022; 376: e064345.

© 2022 Fan JQ et al. *JAMA Network Open*

12. Cohen JA. A Coefficient of Agreement for Nominal Scales. Educational and Psychological Measurement 1960; 20(1): 37--46.

13. Schulz K, Altman D, Moher D. CONSORT 2010 statement: updated guidelines for reporting parallel group randomized trials. Annals of internal medicine 2010; 152(11): 726-32.

14. Ma B, Chen Z, Xu J, Wang Y, Chen K, Ke F, et al. Do the CONSORT and STRICTA Checklists Improve the Reporting Quality of Acupuncture and Moxibustion Randomized Controlled Trials Published in Chinese Journals? A Systematic Review and Analysis of Trends. PloS one 2016; 11(1): e0147244.

15. Linde K, Witt C, Streng A, Weidenhammer W, Wagenpfeil S, Brinkhaus B, et al. The impact of patient expectations on outcomes in four randomized controlled trials of acupuncture in patients with chronic pain. Pain 2007; 128(3): 264-71.

16. Liu X, Xie X, Li Y, Li M, Wang Y, Wang N, et al. Efficacy of manual acupuncture versus placebo acupuncture for generalized anxiety disorder (GAD) in perimenopause women: study protocol for a randomized controlled trial. Trials 2021; 22(1): 833.

© 2022 Fan JQ et al. *JAMA Network Open*
