# Supplementary material for: Effectiveness of Acupuncture for Anxiety Among Patients With Parkinson Disease: A Randomized Clinical Trial
Source: JAMA Netw Open. 2022 Sep 21;5(9):e2232133. doi: 10.1001/jamanetworkopen.2022.32133 (PMC9494193; doi:10.1001/jamanetworkopen.2022.32133)
Supplement: Supplement 3. — Data Sharing Statement [file jamanetwopen-e2232133-s003.pdf]

Data Sharing Statement

Fan JQ, Lu WJ, Tan WQ, et al. Effectiveness of acupuncture for anxiety among patients with Parkinson disease: a randomized clinical trial. *JAMA Netw Open*. 2022;5(9):e2232133. doi:10.1001/jamanetworkopen.2022.32133

Data
Data available: No

Additional Information
Explanation for why data not available: The data that support the findings of this study are available from the corresponding author upon reasonable request.
